# Heart DHA turnover is faster in female compared to male ALA- and EPA-fed mice

**DOI:** 10.1016/j.jlr.2025.100897

**Published:** 2025-09-08

**Authors:** Ruxandra D. Rotarescu, Mahima Mathur, Miranda R. Green, G. Harvey Anderson, Adam H. Metherel

**Affiliations:** Department of Nutritional Sciences, Temerty Faculty of Medicine, University of Toronto, Toronto, Ontario, Canada

**Keywords:** sex differences, DHA turnover, extrahepatic, kinetics, dietary fat, nutrition, omega-3 fatty acids, polyunsaturated fatty acid, fatty acid metabolism

## Abstract

Young females have higher circulating docosahexaenoic acid (DHA) levels than males, though the metabolic basis remains incompletely understood. Building on previous findings that demonstrate higher hepatic synthesis of the DHA precursor, docosapentaenoic acid (DPAn-3), in males, this study extends the investigation to n-3 PUFA turnover in extrahepatic tissues of male and female C57BL/6N mice using compound-specific isotope analysis (CSIA). Animals were fed a 12-week diet enriched in either α-linolenic acid (ALA), eicosapentaenoic acid (EPA), or DHA, starting with a 4-week phase containing low carbon-13 (δ^13^C)-n-3 PUFA, followed by an 8-week phase with high δ^13^C-n-3 PUFA (n = 4 per diet, time point, sex). Heart, perirenal adipose tissue (PRAT), brain, and red blood cells (RBCs) were collected at baseline and at seven time points (1–56 days) post-diet switch, with δ^13^C-n-3 PUFA values modeled by one-phase exponential decay. Compared to males, females exhibited slower turnover of ALA (48%–61% slower) and DPAn-3 (26%–73% slower) from dietary ALA or EPA in the heart, PRAT, and RBCs, resulting from longer half-lives and/or lower DPAn-3 concentrations. Conversely, females showed 26%–28% faster heart DHA turnover from dietary ALA or EPA, despite similar half-lives between sexes. Notably, sex-specific differences in DHA turnover were present only in the heart, whereas DPAn-3 turnover varied across multiple tissues, suggesting a heart-specific mechanism that enhances DHA metabolism in females under low DHA intake. Further research is needed to investigate the physiological significance of these metabolic differences and their potential health implications.

Given the essential roles of long-chain omega-3 (n-3) polyunsaturated fatty acids (PUFA), particularly eicosapentaenoic acid (EPA; 20:5n-3) and docosahexaenoic acid (DHA, 22:6n-3), in cardiovascular and brain health (1, 2) and the known sex differences in cardiovascular disease risk (3), there is a need to elucidate the metabolic factors contributing to these disparities. EPA and DHA have been widely studied for their cardiometabolic effects, including regulation of blood lipids, blood pressure, heart rate, and endothelial function (4). More recently, n-3 PUFAs have been identified as precursors to specialized pro-resolving mediators (SPMs), endogenous lipid mediators that actively promote inflammation resolution and restore tissue homeostasis (5). Assessing n-3 PUFA synthesis and turnover provides insight into the mechanisms underlying sex-specific differences in DHA levels, with potential implications for understanding differential disease risk and guiding sex-specific nutritional recommendations.

DHA is primarily synthesized in the liver from the nutritionally essential α-linolenic acid (ALA; 18:3n-3) and other intermediate precursors such as EPA (6). However, the turnover of DHA and other n-3 PUFA in extrahepatic tissues following hepatic synthesis and distribution, particularly in relation to sex differences, remains unexplored. Previous estimates of DHA synthesis rates have typically relied on plasma fatty acid measures (7, 8, 9) and single-dose tracer approaches (10), both of which have limitations in capturing endogenous n-3 PUFA synthesis and turnover from dietary n-3 PUFA precursors. Given this, and their important functions, understanding the extent to which n-3 PUFA is acquired, whether through direct dietary intake or endogenous synthesis from n-3 PUFA precursors, is critical.

Compound-specific isotope analysis (CSIA) overcomes many of the limitations of previous models, allowing for precise tissue-specific quantification of n-3 PUFA synthesis and turnover rates (11), and has been successfully applied in multiple studies (12, 13, 14, 15, 16). Previously, we demonstrated that while liver DHA synthesis from dietary ALA or EPA did not differ by sex, synthesis of docosapentaenoic acid (DPAn-3; 22:5n-3) from dietary EPA was 66% higher in males (15). Furthermore, the percentage of conversion of dietary ALA to DHA in serum was low (0.2%), but consistent with existing literature estimates (17, 18); however, we showed that DHA conversion was nearly 50-times higher when considering the whole body, suggesting that reliance on serum measures alone may underestimate true synthesis rates (15). These findings highlight the importance of tissue-specific analyses and demonstrate the advantage of CSIA in accurately assessing endogenous fatty acid metabolism.

To build on our previous work, the current study objective was to determine n-3 PUFA turnover rates in extrahepatic tissues, including the heart, perirenal adipose tissue (PRAT), brain, and red blood cells (RBCs), using a mouse model to provide a more comprehensive understanding of sex differences in n-3 PUFA distribution and metabolism throughout the body. We hypothesized that sex-specific differences in DHA levels are influenced by differential metabolic handling of its precursors. To investigate this, we quantified the synthesis and turnover of DHA and its precursors (ALA, EPA) across multiple tissues and in circulation. Our findings revealed that males exhibited faster DPAn-3 turnover in the heart, PRAT, and RBCs, as is consistent with our previous work showing higher hepatic DPAn-3 turnover in males. Notably, however, females demonstrated faster DHA turnover in the heart, a pattern not observed in the liver, suggesting tissue-specific and sex-specific regulation of DHA metabolism. This unique finding raises important questions about the role of DHA metabolism in the heart and its relevance to sex differences in cardiovascular physiology and disease risk.

## Materials and methods

### Animals

All experimental procedures adhered to the guidelines of the Canadian Council on Animal Care and the Regulations of Animals Research Act of Ontario. The study protocol (AUP #20012816) was approved by the Animal Ethics Committee at the University of Toronto. A total of 168 non-littermate C57BL/6N mice (21–28 days old) were obtained from Charles River Laboratories (St. Constant). Upon arrival, the mice were immediately placed on a custom AIN-93G pelleted diet (Dyets Inc). The animal facility maintained standard conditions, including a temperature-controlled environment (21°C) and a 12-h light/dark cycle. Throughout the study, mice were housed in groups of four, provided food and water ad libitum, and handled with care to minimize stress and discomfort.

### Study design

Male and female mice (n = 84 per sex, aged 21–28 days) were assigned to one of three 12-week dietary interventions in which total n-3 PUFA levels were constant, but the carbon-13 isotope content (δ^13^C) of ALA, EPA, or DHA was altered. Each diet consisted of a 4-week low δ^13^C pre-switch phase followed by an 8-week high δ^13^C post-switch phase. For each diet group, 4 different mice per sex were humanely euthanized at each of the seven time points following the dietary switch (0, 1, 3, 7, 14, 28, and 56 days), resulting in 28 mice per sex per diet. (supplemental Fig. S1). At each time point, mice were anesthetized with isoflurane, and blood was collected from the left ventricle. Intracardiac perfusion with cold phosphate-buffered saline was then performed to eliminate potential δ^13^C contamination in tissues from circulating blood. Following perfusion, the heart, PRAT, and brain tissues used in the current study were collected to assess tissue-specific turnover of DHA and other n-3 PUFAs. Blood collected from the left ventricle immediately before perfusion was transferred to microcentrifuge tubes without the addition of anticoagulants and centrifuged at 2000 × *g* for 10 min at 4 °C to separate serum from RBCs. All tissue and blood samples were immediately transferred to dry ice and stored at −80°C until analysis. Fatty acid composition and δ^13^C were quantified using gas chromatography-flame ionization detection (GC-FID) and gas chromatography-isotope ratio mass spectrometry (GC-IRMS), respectively, to determine n-3 PUFA turnover rates. Turnover analyses were based on the time-course data, using n = 4 mice per time point to model the change in δ^13^C enrichment over time and estimate a single half-life with a 95% confidence interval. In contrast, fatty acid concentration and relative percent data were averaged across all 28 animals per group and reflect overall levels during the post-switch period.

### Diet composition and Carbon-13 Signatures (δ^13^C)

The 3 dietary interventions contained 10% fat by weight and were formulated using custom AIN-93G pelleted diets (Dyets Inc.). Each diet differed only in δ^13^C-n-3 PUFA composition between the pre-switch (low δ^13^C) and post-switch (high δ^13^C) phases and provided approximately 2% ALA, 1% EPA, or 1% DHA by weight in total fat (Table 1).

**Table 1:** Fatty acid composition and δ^13^C of the pre- and post-switch ALA, EPA and DHA diets

Fatty Acid	ALA Diets		EPA Diets		DHA Diets	
	Pre-switch	Post-switch	Pre-switch	Post-switch	Pre-switch	Post-switch
SFAs	60.3 ± 1.27	62.2 ± 0.27	58.4 ± 0.67	60.5 ± 0.92	60.6 ± 0.11	62 ± 0.34
MUFAs	6.6 ± 0.21	6.3 ± 0.05	8.1 ± 0.13	7.8 ± 0.18	7.7 ± 0.08	7.2 ± 0.06
n-6 PUFA	30.2 ± 1.03	28.7 ± 0.22	31.3 ± 0.51	29.8 ± 0.7	29.5 ± 0.04	28.6 ± 0.22
18:3n-3, ALA	2.1 ± 0.09	2.1 ± 0.02	n.d	n.d	n.d	n.d
*δ*^*13*^*C-18:3*n*-3*	***−29.8±1.4***	***+9.3±0.5***	--	--	--	--
20:5n-3, EPA	n.d	n.d	1.4 ± 0.03	1.1 ± 0.05	n.d	n.d
*δ*^*13*^*C-20:5*n*-3*	--	--	***−23.8±1.1***	***+10.5±0.4***	--	--
22:6n-3, DHA	n.d	n.d	n.d	n.d	1.4 ± 0.14	1.4 ± 0.19
*δ*^*13*^*C-22:6*n*-3*	--	--	--	--	***−27.7±0.2***	***−10.6±1.4***
n-3 PUFA	2.1 ± 0.09	2.2 ± 0.02	1.4 ± 0.04	1.2 ± 0.05	1.5 ± 0.15	1.4 ± 0.2

ALA, α-linolenic acid; DHA, docosahexaenoic acid; EPA, eicosapentaenoic acid; SFA, saturated fatty acid; MUFA, monounsaturated fatty acid; PUFA, polyunsaturated fatty acid; δ^13^C, carbon-13 content. n.d. – not detected. Fatty acid levels are reported as % weight in total fatty acids and δ^13^C are reported as milliUrey (mUr). All values expressed as means ± SEM (n = 3). Bold italic values indicate δ^13^C.

Dietary levels of ALA, EPA, and DHA were formulated to approximate typical human ALA intake and supplemented doses of EPA and DHA. Using scaling methods that adjust for body surface area discrepancies between species, dietary intake estimates in mice translate to human-equivalent doses of approximately 1,140 mg/day for ALA and 570 mg/day for EPA or DHA. (19). Accordingly, the ALA dose aligns with the usual dietary intake range in humans (1000–2000 mg/day), while the EPA and DHA doses reflect levels achievable through taking a single high-purity supplement capsule daily.

### Lipid extraction and derivatization

Lipids were extracted from 50 mg of pulverized heart and brain tissue and 20 mg of PRAT using a modified Folch method (20). Samples were vortexed in 3 ml of a 2:1 chloroform-methanol solution, with docosatrienoic acid (22:3n-3) ethyl ester added as an internal standard for fatty acid quantification (25 μg for heart and brain, 50 μg for PRAT; Nu-Chek Prep, Inc.). Following the addition of 0.88% potassium chloride, samples were inverted twice and centrifuged to facilitate phase separation. The lipid-containing chloroform phase was then pipetted and isolated as the total lipid extract (TLE). An aliquot of the tissue TLE was dried under a nitrogen stream and then transmethylated using 14% boron trifluoride (BF_3_) in methanol to yield fatty acid methyl esters (FAMEs) (21), as previously described (13). For RBCs, 25 mg were directly transesterified with 14% BF_3_ and hexane containing 5 μg of docosatrienoic acid (22:3n-3) ethyl ester. The resulting hexane layer containing FAMEs was dried under nitrogen and reconstituted in 100 μl of heptane for tissues and 75 μl for red blood cells to facilitate transfer to GC vials for subsequent analysis by GC-FID and GC-IRMS, as previously described (22).

### Fatty acid quantification by GC-flame ionization detection (GC-FID)

FAMEs were analyzed on a Varian 430 GC-FID (SCION Instruments, Goes, NL) equipped with a DB-FFAP 30 m × 0.25 mm i.d. × 0.25 μm film thickness, nitroterephthalic acid modified, polyethylene glycol, capillary column (Agilent Technologies), as previously described (16). Peak retention times were identified by comparison to an external FAME standard (GLC-462, Nu-Chek Prep, Inc.). Fatty acid concentrations were quantified relative to the known amount of 22:3n-3 ethyl ester in the sample. Following GC-FID, vials were re-capped and stored at −80°C until further analysis by GC-IRMS for CSIA, as previously described (22).

### Fatty acid ^13^C Signature (δ^13^C) determination by GC-IRMS

CSIA of isolated FAMEs was performed using GC-IRMS, as previously described (22). FAMEs were injected in splitless mode via a TriPlus RSH autosampler (Thermo Scientific) onto a Supelco SP-2560 capillary column (Merck & Co., Inc., Rahway, NJ) installed in a Trace 1310 GC (Thermo Scientific). Certified, calibrated 20-carbon FAME reference materials—USGS70, USGS71, and USGS72 (United States Geological Survey)— were alternately injected approximately every 10 samples within each programmed sequence. Linear regression of measured versus true values (−30.53 ± 0.04 mUr, −10.50 ± 0.03 mUr, and −1.54 ± 0.03 mUr for USGS70, USGS71, and USGS72, respectively) was used to generate a normalization equation for calculating true δ^13^C values.

### Kinetics equations

Heart, PRAT, brain and RBC δ^13^C values following the dietary switch were plotted over the seven time points and modeled using a one-phase exponential decay function to generate a curve of best-fit, where *y*_*0*_ represents the curve at time zero, the plateau corresponds to the value at infinite time, and *k* is the rate constant (determined from Equation 1). Half-lives (*t*_*1/2*_) were calculated from the rate constants of the best-fit curves using Equation 2. Turnover rates were estimated based on the rate of loss (*J*_*out*_, nmol/g/day for RBCs, μmol/g/day for tissues) using Equation 3, where *C*_*FA*_ represents the mean n-3 PUFA concentration during the study (Equation 2). As fatty acids are degraded and replaced, the model estimates half-lives by tracking the incorporation of δ^13^C from dietary DHA or its precursors (ALA, EPA) into newly synthesized n-3 PUFAs within the lipid pool. Notably, the assumption that the calculated net rate of DHA metabolic loss (*J*_*out*_) equals the net rate of DHA incorporation relies on maintaining a steady-state lipid pool throughout the study. This was ensured by formulating the pre-switch and post-switch diets to contain the same amounts of ALA, EPA, or DHA, with the only difference being the δ^13^C enrichment of the n-3 PUFA oil used. This approach allowed us to assess turnover without altering total dietary n-3 PUFA intake.

Eq. 1 $$y={\left(y_{0}-plateau\right)}^{-kx}+plateau$$

Eq. 2 $$t_{1/2}=\frac{\ln\;2}{\kappa }$$

Eq. 3 $$J_{out}=\frac{0.693C_{FA}}{t_{1/2}}$$

### Statistics

All statistical analyses were conducted using GraphPad Prism 10.0.2 (GraphPad Software Incorp., California, USA) with significance set at *P* < 0.05. Data are presented as means ± SEM. Sex differences in mean n-3 PUFA levels (concentration and relative %) and kinetic values were assessed using independent t-tests with Welch correction within each dietary group. Multiple *t* tests were conducted without correction, as these represent exploratory analyses in tissues beyond the previously published primary outcome (15). Kinetic modeling, including half-lives (Equation 2) and turnover rates (Equation 3), was performed using one-phase exponential decay dissociation curves. To assess significance, 95% confidence intervals were converted to standard deviations by dividing the confidence interval width by the t-statistic corresponding to the probability and degrees of freedom, then multiplying by the square root of the sample size (n) (Equation 5). This calculation follows the process outlined in Section 6.5.2.2 of *The Cochrane Handbook for Systematic Reviews of Interventions* (23).

Equation 5 $$SD=\sqrt{n}\times \frac{\left(upper\;limit-lower\;limit\right)}{t-statistic\times \#\;of\;groups}$$

## Results

### Heart, perirenal adipose tissue (PRAT), brain, and red blood cell (RBC) n-3 PUFA levels

**Fig. 1:**
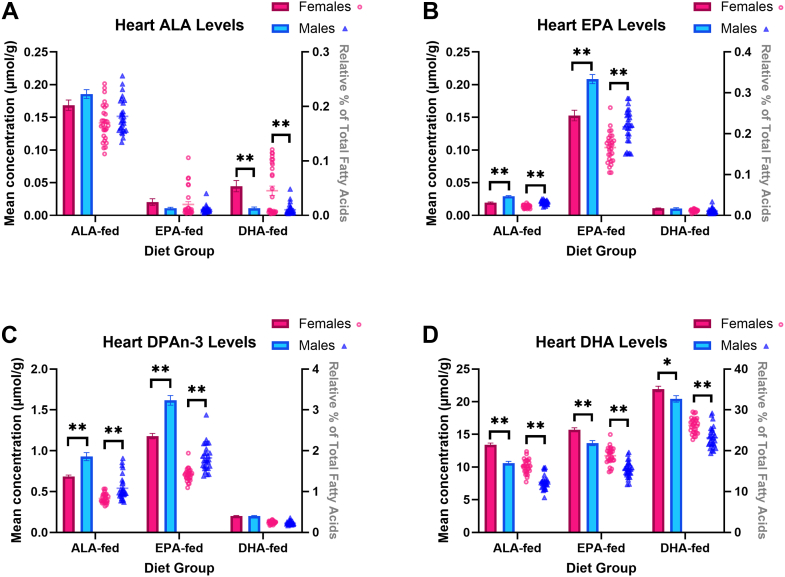
Mean heart concentrations (μmol/g, bar graph) and relative percent (fatty acid in % total fatty acids, scatterplot) of (A) ALA, (B) EPA, (C) DPAn-3, and (D) DHA of ALA-, EPA-, and DHA-fed mice. ∗ and ∗∗ represent statistically significant differences relative to females within each diet group as determined by an independent *t* test at *P*-values < 0.05 and < 0.001, respectively. Values are means ± SEM (n = 28). ALA, α-linolenic acid; DHA, docosahexaenoic acid; DPAn-3, docosapentaenoic acid; EPA, eicosapentaenoic acid.

**Fig. 2:**
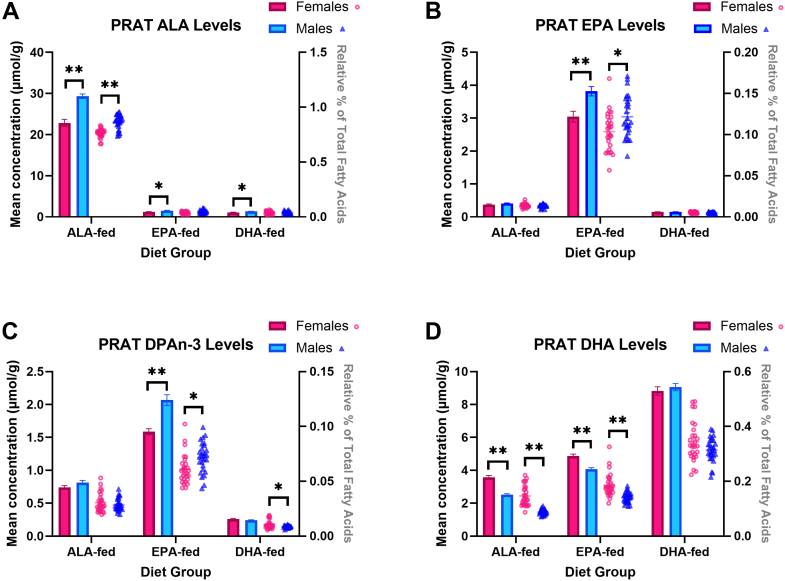
Mean perirenal adipose tissue (PRAT) concentrations (nmol/ml, bar graph) and relative percent (fatty acid in % total fatty acids, scatterplot) of (A) ALA, (B) EPA, (C) DPAn-3, and (D) DHA of ALA-, EPA-, and DHA-fed mice. ∗ and ∗∗ represent statistically significant differences relative to females within each diet group as determined by an independent *t* test at *P*-values < 0.05 and < 0.001, respectively. Values are means ± SEM (n = 28). ALA, α-linolenic acid; DHA, docosahexaenoic acid; DPAn-3, docosapentaenoic acid; EPA, eicosapentaenoic acid.

**Fig. 3:**
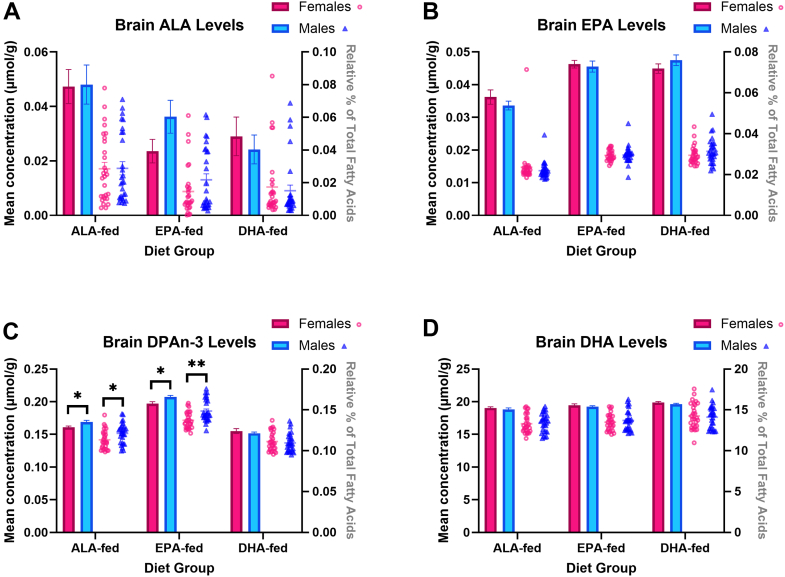
Mean brain concentrations (μmol/g, bar graph) and relative percent (fatty acid in % total fatty acids, scatterplot) of (A) ALA, (B) EPA, (C) DPAn-3, and (D) DHA of ALA-, EPA-, and DHA-fed mice. ∗ and ∗∗ represent statistically significant differences relative to females within each diet group as determined by an independent *t* test at *P*-values < 0.05 and < 0.001, respectively. Values are means ± SEM (n = 28). ALA, α-linolenic acid; DHA, docosahexaenoic acid; DPAn-3, n-3 docosapentaenoic acid; EPA, eicosapentaenoic acid.

**Fig. 4:**
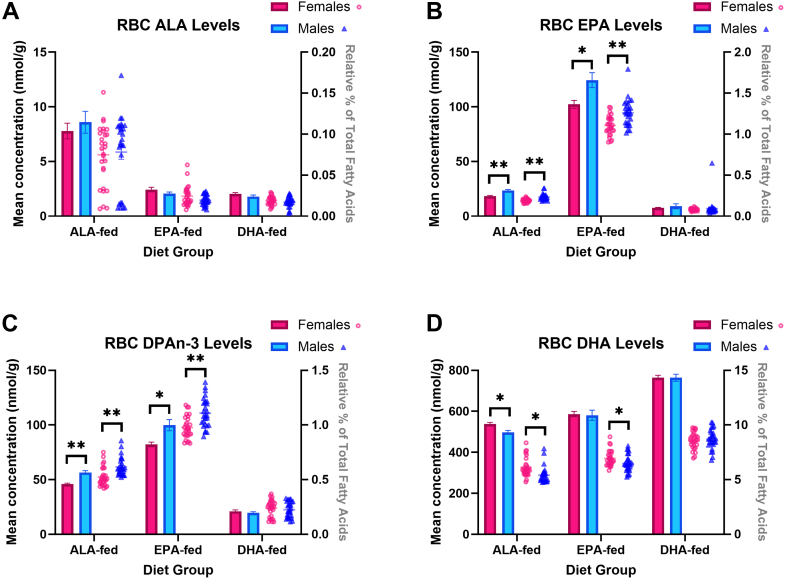
Mean red blood cell (RBC) concentrations (nmol/g, bar graph) and relative percent (fatty acid in % total fatty acids, scatterplot) of (A) ALA, (B) EPA, (C) DPAn-3, and (D) DHA of ALA-, EPA-, and DHA-fed mice. ∗ and ∗∗ represent statistically significant differences relative to females within each diet group as determined by an independent *t* test at *P*-values < 0.05 and < 0.001, respectively. Values are means ± SEM (n = 28). ALA, α-linolenic acid; DHA, docosahexaenoic acid; DPAn-3, n-3 docosapentaenoic acid; EPA, eicosapentaenoic acid.

N-3 PUFA levels (concentration and relative %) in the heart (Fig. 1), PRAT (Fig. 2), the brain (Fig. 3), and RBC (Fig. 4) were measured. Additionally, sex-specific differences in the heart (Supplemental Table 1 & 2), PRAT (supplemental Tables S3 and S4), brain (supplemental Tables S5 and S6), and RBC (supplemental Tables S7 and S8) fatty acid concentrations and relative % were also determined.

### Heart n-3 PUFA levels

Heart ALA concentrations (μmol/g ± SEM) were not significantly different between sexes in ALA- or EPA-fed animals. Heart EPA concentrations were 31% lower in ALA-fed females (*P* < 0.0001) and 26% lower in EPA-fed females (*P* < 0.0001), with the % EPA mirrored these patterns. Heart DPAn-3 concentrations were significantly lower in females than in males in the ALA-fed (26% lower, *P* < 0.0001) and EPA-fed (27% lower, *P* < 0.0001) groups, and was again matched by nearly identical findings for % DPAn-3Heart DHA concentrations (and relative %) were significantly higher in females compared to males across all dietary groups: 27% higher in the ALA-fed (*P* < 0.0001), 15% higher in the EPA-fed (*P* = 0.0001), and 7.4% higher in the DHA-fed group (*P* = 0.03).

### Perirenal adipose tissue (PRAT) n-3 PUFA levels

PRAT ALA concentrations (μmol/g ± SEM) were 22% lower in females compared to males in the ALA-fed group (*P* < 0.0001), matched by a 12% lower % ALA (*P* < 0.0001) PRAT EPA concentrations were 20% lower in EPA-fed females (*P* = 0.0008), while relative percent was 15% lower (*P* = 0.007); no sex differences were found in other groups. PRAT DPAn-3 concentrations were 23% lower in EPA-fed females (*P* < 0.0001), but not significantly different in the ALA- or DHA-fed groups, while % DPAn-3 was 15% lower in EPA-fed females (*P* = 0.004) but 23% higher in DHA-fed females (*P* = 0.01), with no difference in the ALA-fed group. PRAT DHA concentrations were 42% higher in ALA-fed females (*P* < 0.0001) and 20% higher in EPA-fed females (*P* < 0.0001), with no significant difference in the DHA-fed group. Finally, % DHA followed a similar trend: 67% higher in ALA-fed (*P* < 0.0001) and 32% higher in EPA-fed females (*P* < 0.0001), with no sex difference in DHA-fed animals.

### Brain n-3 PUFA levels

Brain DPAn-3 concentrations were significantly lower in females: 4.8% lower in ALA-fed (*P* = 0.01) and 5.0% lower in EPA-fed mice (*P* = 0.01). Similarly, % DPAn-3 was 6.5% lower in ALA-fed (*P* = 0.02) and 7.6% lower in EPA-fed (*P* = 0.0007) females. Brain concentrations (μmol/g ± SEM) and % of ALA, EPA, and DHA in the brain did not differ significantly between sexes in any dietary group.

### Red blood cells (RBC) n-3 PUFA levels

RBC ALA concentrations and % ALA were not significantly different between sexes in the ALA-fed group. However, RBC EPA concentrations were 18% lower in EPA-fed (*P* = 0.006) females relative to males, while % EPA was 12% lower in EPA-fed females (*P* = 0.0002). RBC DPAn-3 concentrations were lower in females: 19% lower in ALA-fed (*P* < 0.0001) and 17% lower in EPA-fed groups (*P* = 0.003), with parallel differences observed in % DPAn-3RBC DHA concentrations were 8.1% higher in ALA-fed females (*P* = 0.002), with no significant sex difference in the EPA- or DHA-fed groups. Finally, % DHA was higher in ALA-fed (12.8%, *P* = 0.002) and EPA-fed females (7.2%, *P* = 0.02), but not in the DHA-fed group.

### N-3 PUFA rate constants (k), half-lives (t_1/2_), and turnover rates

One-phase exponential decay modeling (supplemental Figs. S2–S5) was used to calculate rate constants (k, days^-1^ × 10^-3^), half-lives (t_1_/_2_, days), and turnover rates in the heart (Table 2), PRAT (Table 3), and RBCs (Table 4) of ALA-, EPA-, and DHA-fed mice. Independent t-tests (*P* < 0.05) were used to assess sex differences. Due to the limited study duration of 56 days, only DHA-fed kinetic modeling was possible, and yet the expected δ^13^C plateau was not reached. However, to provide transparency and additional context, detailed brain kinetic data have been provided in supplemental Table S9.

**Table 2:** Heart n-3 PUFA turnover kinetics in ALA-, EPA- and DHA-fed male and female mice

Diet Group	Measured n-3 PUFA	Rate constant (*k*) (days^−1^ × 10^−3^)		Half-life (*t*_*1/2*_) (days)		Turnover Rate (*J*_*out*_) (μmol/g/d)	
		Female	Male	Female	Male	Female	Male
ALA-fed	ALA	0.398 ± 0.080	0.923 ± 0.160^a^	1.74 ± 0.37	0.751 ± 0.130^a^	0.067 ± 0.010	0.171 ± 0.030^a^
	DPAn-3	0.054 ± 0.004	0.075 ± 0.004^a^	13.0 ± 1.0	9.31 ± 0.50^a^	0.037 ± 0.003	0.069 ± 0.004^a^
	DHA	0.031 ± 0.002	0.031 ± 0.002	22.4 ± 1.3	22.8 ± 1.9	0.410 ± 0.020	0.320 ± 0.030^a^
EPA-fed	EPA	0.192 ± 0.018	0.203 ± 0.024	3.62 ± 0.34	3.42 ± 0.41	0.029 ± 0.003	0.042 ± 0.005
	DPAn-3	0.090 ± 0.005	0.089 ± 0.005	7.73 ± 0.50	7.78 ± 0.50	0.106 ± 0.006	0.144 ± 0.009^a^
	DHA	0.044 ± 0.003	0.040 ± 0.001	15.9 ± 1.0	17.5 ± 0.6	0.680 ± 0.040	0.540 ± 0.020^a^
DHA-fed	DHA	0.067 ± 0.003	0.068 ± 0.002	10.3 ± 0.5	10.1 ± 0.3	1.47 ± 0.07	1.40 ± 0.04

All values are expressed as means ± SEM (n = 4 mice per diet, per time point, per sex). ALA, α-linolenic acid; DHA, docosahexaenoic acid; DPAn-3, docosapentaenoic acid; EPA, eicosapentaenoic acid.

a represents statistically significant differences relative to females within each n-3 PUFA kinetic parameter, as determined by independent *t* test, *P*-value < 0.05.

**Table 3:** Perirenal adipose tissue (PRAT) n-3 PUFA turnover kinetics of ALA-, EPA- and DHA-fed male and female mice

Diet Group	Measured n-3 PUFA	Rate constant (*k*) (days^−1^ × 10^−3^)		Half-life (*t*_*1/2*_) (days)		Turnover Rate (*J*_*out*_) (μmol/g/d)	
		Female	Male	Female	Male	Female	Male
ALA-fed	ALA	0.053 ± 0.008	0.079 ± 0.004^a^	13.1 ± 2.0	8.75 ± 0.47	1.21 ± 0.17	2.32 ± 0.12^a^
	EPA	0.094 ± 0.035	0.103 ± 0.031	7.34 ± 2.70	6.73 ± 2.00	0.035 ± 0.013	0.042 ± 0.013
	DPAn-3	0.038 ± 0.005	0.073 ± 0.005^a^	18.4 ± 3.0	9.5 ± 0.6^a^	0.028 ± 0.004	0.059 ± 0.004^a^
	DHA	0.025 ± 0.003	0.039 ± 0.003^a^	27.6 ± 3.7	18.0 ± 1.5	0.089 ± 0.011	0.097 ± 0.008
EPA-fed	EPA	0.095 ± 0.02	0.086 ± 0.008	7.29 ± 1.70	8.10 ± 0.76	0.290 ± 0.06	0.326 ± 0.03
	DPAn-3	0.061 ± 0.007	0.095 ± 0.01^a^	11.3 ± 1.4	7.28 ± 0.70^a^	0.097 ± 0.01	0.20 ± 0.02^a^
	DHA	0.044 ± 0.004	0.058 ± 0.006	15.7 ± 1.6	11.9 ± 1.4	0.21 ± 0.02	0.24 ± 0.03
DHA-fed	DHA	0.064 ± 0.006	0.078 ± 0.007	10.8 ± 1.1	8.90 ± 0.80	0.57 ± 0.06	0.70 ± 0.06

All values are expressed as means ± SEM (n = 4 mice per diet, per time point, per sex). ALA, α-linolenic acid; DHA, docosahexaenoic acid; DPAn-3, docosapentaenoic acid; EPA, eicosapentaenoic acid.

a represents statistically significant differences relative to females within each n-3 PUFA kinetic parameter, as determined by independent *t* test, *P*-value < 0.05.

**Table 4:** Red blood cell (RBC) n-3 PUFA turnover kinetics of ALA-, EPA- and DHA-fed male and female mice

Diet Group	Measured n-3 PUFA	Rate constant (*k*) (days^−1^ × 10^−3^)		Half-life (*t*_*1/2*_) (days)		Turnover Rate (*J*_*out*_) (nmol/g/d)	
		Female	Male	Female	Male	Female	Male
ALA-fed	EPA	0.053 ± 0.009	0.072 ± 0.013	13.2 ± 2.6	9.6 ± 1.8	0.96 ± 0.17	1.7 ± 0.30
	DPAn-3	0.012 ± 0.002	0.034 ± 0.004^a^	60.1 ± 13.8	20.3 ± 2.5^a^	0.53 ± 0.09	1.94 ± 0.22^a^
	DHA	0.017 ± 0.002	0.017 ± 0.003	41.0 ± 3.8	40.7 ± 7.3	9.10 ± 0.79	8.48 ± 1.23
EPA-fed	EPA	0.070 ± 0.009	0.114 ± 0.014^a^	10.0 ± 1.4	6.1 ± 0.75^a^	7.11 ± 0.93	14.1 ± 1.73^a^
	DPAn-3	0.020 ± 0.003	0.033 ± 0.003^a^	34.0 ± 4.9	21.4 ± 2.4	1.67 ± 0.21	3.24 ± 0.33^a^
	DHA	0.026 ± 0.002	0.030 ± 0.002	26.5 ± 2.1	23.1 ± 1.9	15.3 ± 1.13	17.5 ± 1.35
DHA-fed	DHA	0.030 ± 0.002	0.035 ± 0.004	23.4 ± 2.0	20.0 ± 2.7	22.7 ± 1.83	26.5 ± 3.18

All values are expressed as means ± SEM (n = 4 mice per diet, per time point, per sex). ALA, α-linolenic acid; DHA, docosahexaenoic acid; DPAn-3, docosapentaenoic acid; EPA, eicosapentaenoic acid.

a represents statistically significant differences relative to females within each n-3 PUFA kinetic parameter, as determined by independent *t* test, *P*-value < 0.05.

### Heart n-3 PUFA k, t_1/2_, and turnover

ALA and DPAn-3 rate constants were 57% and 28% lower, and half-lives 132% and 14% longer (*P* < 0.05) in females than in males, respectively, while DHA rate constants and half-lives did not differ in the ALA dietary group. Furthermore, heart ALA and DPAn-3 turnover rates from dietary ALA were 61% (*P* = 0.02) and 46% (*P* = 0.0005) slower in females, respectively, whereas DHA turnover from dietary ALA was 28% higher in females compared to males (*P* = 0.04). In EPA-fed mice, no sex differences were observed for the DHA rate constant or half-life; however, DPAn-3 turnover from dietary EPA was 26% slower in females than in males (*P* = 0.01). Conversely, DHA turnover rate was 26% higher in females compared to in males (*P* = 0.03). No sex differences in DHA-fed animals were determined.

### Perirenal adipose tissue (PRAT) n-3 PUFA k, t_1/2_, and turnover

In ALA-fed mice, ALA, DPAn-3, and DHA rate constants were 33%, 48%, and 35% lower in females. No significant sex differences in ALA (*P* = 0.08), EPA (*P* = 0.86), or DHA (*P* = 0.053) half-lives were observed; however, DPAn-3 half-life in PRAT was 94% longer in females (*P* = 0.03). ALA and DPAn-3 turnover rates from dietary ALA were 48% (*P* = 0.002) and 53% (*P* = 0.001) slower in females compared to in males, while EPA and DHA turnover rates did not differ by sex. In EPA-fed animals, the DPAn-3 rate constant was 36% lower (*P* = 0.0025), the half-life was 55% longer (*P* = 0.04) in females, and accordingly, the turnover rate was 52% slower in females compared to males (*P* = 0.005). In DHA-fed mice, no significant sex differences were found for any PRAT kinetic parameters.

### Red blood cell (RBC) n-3 PUFA k, t_1/2_ and turnover

The DPAn-3 rate constant in RBCs was 66% lower in females than in males in the ALA diet group (*P* = 0.002). Similarly, RBC DPAn-3 half-lives in females were 196% longer than in males (*P* = 0.03), and DPAn-3 turnover rates from dietary ALA were 73% lower in females than in males (*P* = 0.001). No significant differences were observed for EPA or DHA rate constants or half-lives. In EPA-fed animals, females had 39% and 37% lower EPA and DPAn-3 rate constants, respectively, compared to males. EPA half-life was 64% longer in females than in males (*P* = 0.047), with no significant differences in DPAn-3 (*P* = 0.058). Consistent with this, EPA and DPAn-3 turnover were 50% (*P* = 0.01) and 48% (*P* = 0.007) lower in females than in males, respectively. No sex differences were identified for any measure of DHA in EPA-fed mice (*P* > 0.05). In DHA-fed animals, no sex differences were observed.

## Discussion

**Fig. 5:**
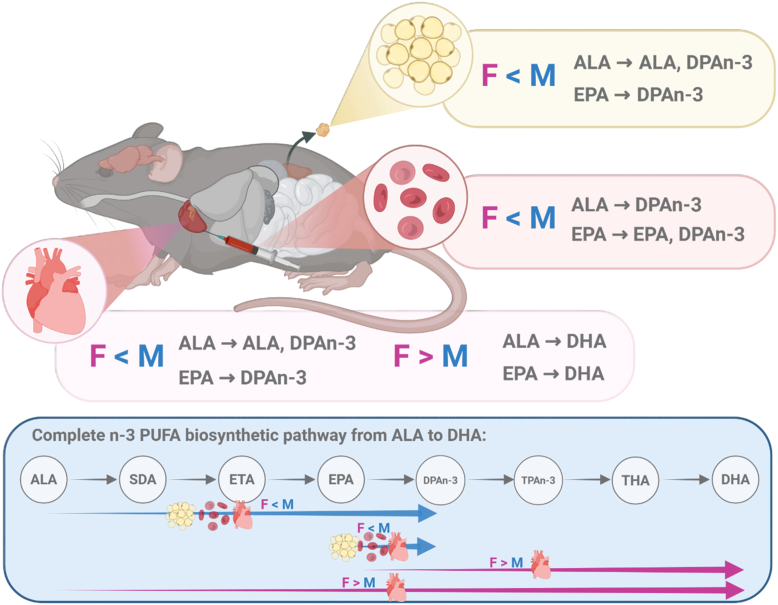
Summary of significant sex differences in the rate of turnover of n-3 PUFA from dietary ALA and EPA in the heart, perirenal adipose tissue, and red blood cells. Created with BioRender.com. ALA, α-linolenic acid; DHA, docosahexaenoic acid; DPAn-3, n-3 docosapentaenoic acid; EPA, eicosapentaenoic acid; F, females; M, males.

Our study reveals that tissue-specific sex differences in n-3 PUFA levels may arise from differences in the rate of synthesis and accumulation of those n-3 PUFA from dietary and metabolic precursors (ALA, EPA). When provided with ALA or EPA alone, females consistently demonstrated slower turnover of DPAn-3 in the heart, PRAT, and RBCs, and faster turnover of DHA in the heart compared to males. These differences were attenuated when DHA was provided directly in the diet. Figure 5 provides a summary of these significant sex- and tissue-specific differences.

The slower DPAn-3 turnover observed in the heart and PRAT of females in the present study is consistent with slower DPAn-3 turnover in the serum of EPA-fed females compared to males (15). However, this trend did not extend to DHA, as while serum DHA turnover was slower in EPA-fed females, heart DHA turnover was faster. Previous work using the same animals found no sex differences in hepatic DHA synthesis or turnover from dietary ALA or EPA (15), suggesting that hepatic synthesis alone cannot explain the differences observed in extrahepatic tissues like the heart. This divergence may indicate that DHA turnover is more strongly influenced by tissue-specific regulatory mechanisms, with DPAn-3 turnover more dependent on availability from circulation. Although fish and standard n-3 PUFA supplements contain DPAn-3, its biological functions and mechanisms of action remain underexplored. DPAn-3 shares structural and functional properties with DHA (24) and serves as a precursor for SPM synthesis, potentially contributing to cardiovascular health (25). Sex-specific differences in lipid mediator profiles have been reported in both rodents (26, 27, 28) and humans (29, 30), with higher plasma EPA- and DHA-derived SPMs observed in younger women, relative to men and older women, implicating sex hormones in lipid mediator biosynthesis (29). However, more research is needed to better understand the role of sex on DPAn-3-derived SPMs.

One possible mechanism for the sex-specific regulation of DHA synthesis and/or retention within the heart tissue resides with *Elovl2*, encoding for the enzyme responsible for the 2-carbon elongations of EPA to DPAn-3 and DPAn-3 to TPAn-3, which contains an estrogen response element and is upregulated by estradiol (31). While prior studies have reported no detectable expression of *Elovl2* in rodent hearts (32, 33), the studies were conducted exclusively in male rodents, raising the possibility that Elovl2 expression/activity may be present in females. However, while *Elovl2* mRNA was reported to be 1.1-fold higher in the livers of female rats compared to males, they also reported heart *Elovl2* mRNA expression and protein content, with no sex differences observed (34). Sex-specific differences in ELOVL2 enzyme activity, tissue-specific transporter expression/affinity, or phospholipid incorporation efficiency in the heart could also contribute to differing turnover rates; however, further research is needed to investigate these possibilities. Supporting this, female rat hearts are reported to have higher proportions of DHA in phospholipids, but not in triacylglycerols, compared to males (34). Interestingly, a recent study demonstrated that in the heart, females exhibit greater release of DHA, EPA, and ALA than males from phospholipids, suggesting higher phospholipase A_2_ activity toward these fatty acids in females (35). This enzymatic difference may contribute to the faster female heart DHA turnover observed in in our study and represents an interesting path for future investigation within our models.

Our study also revealed sex-specific differences in PRAT turnover of DHA precursors, particularly ALA and DPAn-3. Once considered primarily structural, perirenal adipose tissue (PRAT) is now recognized as a metabolically active depot with regulatory roles in cardio-renal function and has pronounced sex-specific differences in morphology and function (36). Compared to other visceral fat depots, PRAT is more strongly associated with CVD risk and exhibits sexual dimorphism, including greater mass in males, and greater browning capacity and UCP-1 expression in females (36). Our study is the first to examine n-3 PUFA turnover in PRAT by sex, contributing to a largely understudied area with potential cardiovascular implications. A similar tracer-based study previously examined epididymal fat in mice consuming diets higher in DHA (2% vs. our 1% in total fat) (37). Unlike PRAT, which undergoes brown-to-white adipocyte transition, epididymal fat represents a classical white adipose depot. Although this previous study was informative, potential variability was introduced by switching not only the δ^13^C but also the n-3 PUFA itself (ALA to DHA or *vice-versa*), complicating turnover interpretation. In contrast, we maintained consistent n-3 PUFA in the diets to ensure steady-state conditions and avoid artificially altered turnover (*J*_*out*_) estimates due to changing pool sizes. Despite methodological differences, their reported adipose DHA half-lives (5.4–7.9 days) (37) align well with ours in the DHA-fed group (8.9–10.8 days). Our ALA-fed mice exhibited longer PRAT DHA half-lives (18–27.6 days), demonstrating slower turnover when DHA is endogenously synthesized exclusively from ALA. Similarly, the prior study reported a half-life of 30.1 days in mice switched from DHA to ALA (37), highlighting the sensitivity of adipose DHA turnover to dietary n-3 PUFA source. Notably, it could not be explored in the previous male-only study.

In the brain, the short length of our study compared to previous work (16, 37, 38) likely resulted in significant underestimation of brain half-lives and overestimation of turnover rates, limiting our ability to detect potential sex-specific differences. This is best exemplified by the δ^13^C plateaus generated for our one-phase exponential decay model that were significantly lower (−21.4 and −21.7 mUr in females and males, respectively) than the δ^13^C of the DHA post-switch diet (−10.6 mUr), the δ^13^C value that brain DHA is expected to reach at the plateau. Nevertheless, brain DHA turnover data offer important context, especially when compared to tissues such as the heart, where sex differences in DHA turnover were evident. Consequently, any conclusion regarding brain DHA half-lives and turnover remains preliminary. Future studies investigating sex differences in brain DHA turnover must be designed with an appropriate length for accurate kinetic modeling.

EPA and DHA kinetics vary across blood compartments, with relatively slower membrane turnover (39) making RBCs a preferred biomarker of long-term n-3 PUFA status, such as with the Omega-3 Index, which is a widely used blood biomarker for n-3 PUFA status measuring the % of EPA + DHA in RBCs (40). In our study, the estimated RBC half-lives of DHA-fed mice (23.4 ± 2.0 days in females, 20.0 ± 2.7 days in males) are in close agreement with the reported half-lives for RBC lifespans in C57BL/6 mice (22.4 ± 0.9 days in females, 22.9 ± 1.2 days in males) (41), lending further support to the validity of our turnover estimates and reinforcing the utility of RBCs as a biomarker for long-term n-3 PUFA status compared to our previously reported serum DHA half-lives in DHA-fed females (5.8 ± 0.6 days) and males (4.1 ± 0.3 days) (15). In addition to serving as biomarkers of longer-term fatty acid status, RBCs may also reflect sex-specific regulation of systemic lipid metabolism. RBC fatty acid profiles have been shown to correlate with those of metabolically active tissues such as the heart and brain (42), and sex-specific differences in RBC n-3 PUFA content have been previously reported (43). Moreover, variation in unsaturated fatty acid incorporation may impact RBC membrane fluidity and function (44), supporting the inclusion of RBC analyses in studies investigating sex-specific differences in n-3 PUFA metabolism.

Interestingly, DHA half-lives were shorter in heart and perirenal adipose tissue compared to RBCs. This likely reflects faster membrane lipid turnover in these metabolically active tissues, whereas DHA in RBCs is primarily incorporated during erythropoiesis and remains relatively stable throughout the cell’s lifespan, contributing to a longer apparent half-life. However, evidence suggests that mature RBCs are not entirely static in their lipid composition. Although mature RBCs lack organelles and the enzymatic pathways for de novo lipid synthesis, they retain ATP-dependent, protein-mediated phospholipid transporters that mediate membrane remodeling and may allow for the selective incorporation of fatty acids such as DHA (45). Supporting this, an early in vitro study demonstrated that nonesterified fatty acids can be incorporated into both RBC triglycerides and membrane phospholipids (46). A more recent in vivo tracer study showed that plasma FFAs can be incorporated into RBC triglycerides. Notably, while RBC triglycerides are enriched in saturated fatty acids, unsaturated fatty acids such as DHA are preferentially incorporated into membrane phospholipid pools, crucial for maintaining membrane structure and fluidity (47). Nonetheless, given the agreement between our estimated DHA half-lives and the typical RBC lifespan, it is likely that erythropoiesis is the main determinant of DHA kinetics, rather than remodeling processes in mature RBCs.

Several limitations should be considered when interpreting the current findings. First, mice were not fasted prior to perfusion, which may have introduced variability in δ^13^C levels; however, their nocturnal feeding patterns likely minimized this effect during daytime tissue collections (48). Second, C57BL/6 mice carry a homozygous mutation in the Secretory Group II Phospholipase A2 (sPLA2) gene (49), potentially affecting n-3 PUFA phospholipid metabolism compared to strains like BALB/c. Despite this, our adipose DHA turnover rates are comparable to those reported in BALB/c mice (38). Third, PRAT has a transitional phenotype, initially brown-like but undergoing whitening with age and environmental changes such as cold exposure (36). Since our animals were assessed at sexual maturity, browning is unlikely to have influenced results. Nevertheless, future studies including multiple adipose depots will be important for determining whether the observed effects are depot-specific. Additionally, while the ALA and EPA diets were purposefully deficient in other n-3 PUFAs to isolate the metabolic contributions of these individual precursors, this inherently limits the translational relevance to human dietary patterns, which typically contain mixed n-3 PUFA sources. Finally, while our model quantifies overall DHA turnover, it does not delineate the underlying metabolic pathways driving these differences, such as conversion to lipid mediators and/or β-oxidation.

Overall, our work demonstrates significant sex-specific turnover rates of ALA and its elongation products (EPA, DPAn-3) across multiple extrahepatic tissues when mice were fed ALA- or EPA-only diets. Females consistently demonstrated slower DPAn-3 turnover compared to males; however, heart DHA turnover is faster in females. These findings highlight the importance of considering sex as a biological variable in studies assessing n-3 PUFA metabolism and underscore the value of kinetic approaches in interpreting nutritional interventions and their outcomes. The male sex may be a stronger independent risk factor for cardiovascular disease (CVD) than smoking, diabetes, elevated plasma lipids, or high blood pressure (3). Although no significant interaction between supplement and sex was observed in the EPA monotherapy clinical trials, JELIS and REDUCE-IT, the significantly reduced cardiovascular risk endpoints appeared to be driven primarily by male participants (50, 51). These findings, together with our own findings of faster heart DHA turnover in females of ALA- and EPA-fed mice, raise important questions about the potential link between sex-specific n-3 PUFA metabolism and cardiometabolic risk. Sex-specific differences in DHA turnover were evident in the heart but not in RBCs, perirenal adipose tissue, or brain, indicating tissue-specific regulation. Given that sex differences in DPAn-3 turnover were observed across multiple tissues, but only the heart exhibited differences in DHA turnover, this pattern supports the existence of a heart-specific mechanism that increases DHA metabolism in females when DHA intake is low. Further research is warranted to explore whether the metabolic patterns identified here have implications for sex-related differences in disease risk. As the field of personalized nutrition advances, characterizing metabolic differences by sex and gender may allow for more precise and effective n-3 PUFA dietary recommendations to support health.

## Data availability

Additional data is to be shared upon request from the corresponding author (A. H. M.).

## Supplemental data

This article contains supplemental data.

## Conflict of interest

The authors declare the following financial interests/personal relationships which may be considered as potential competing interests: G. H. A. is the Director of the NSERC University–Industry Collaborative Research Program in Food Safety, Nutrition and Regulatory Affairs at the Department of Nutritional Sciences, Faculty of Medicine, University of Toronto.

A. H. M. is on the Board of Directors of the International Society for the Study of Fatty Acids and Lipids, is a Science Advisor for Benexia and Natures Crops International and was a co-applicant on a joint government/industry funded research grant with Natures Crops International.
