# Supplementary material for: Heart DHA turnover is faster in female compared to male ALA- and EPA-fed mice
Source: J Lipid Res. 2025 Sep 8;66(10):100897. doi: 10.1016/j.jlr.2025.100897 (PMC12519242; doi:10.1016/j.jlr.2025.100897)
Supplement: Supplemental Tables and figures [file mmc1.docx]

**SUPPLEMENTAL INFORMATION:**

**Sex-specific differences in heart, perirenal adipose and red blood cell n-3 PUFA turnover in ALA- and EPA-fed mice**

Ruxandra D. Rotarescu^1^, Mahima Mathur^1^, Miranda R. Green^1^, G. Harvey Anderson^1^, Adam H. Metherel*^1^

^1^Department of Nutritional Sciences, Temerty Faculty of Medicine, University of Toronto, 1 King’s College Circle, Toronto, Ontario, Canada, M5S 1A8


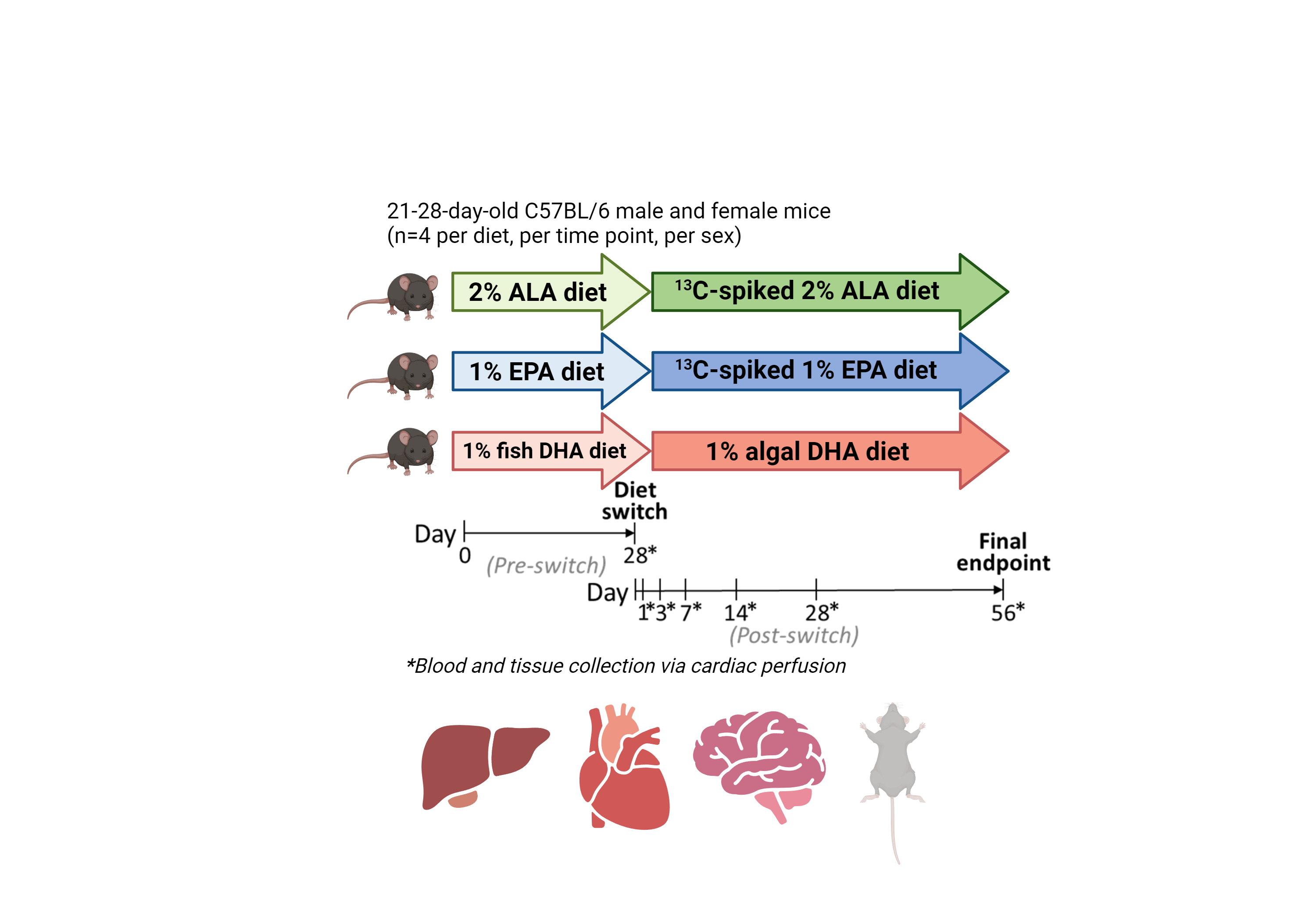


**Supplemental Figure 1** – Study design. ALA, a-linolenic acid; DHA, docosahexaenoic acid; EPA, eicosapentaenoic acid. Created with BioRender.com.

**Supplemental Table 1** – Heart fatty acid concentrations of ALA-, EPA- and DHA-fed female and male mice.

|  | Heart Concentration (µmol/g) | | | | | | | | |
| --- | --- | --- | --- | --- | --- | --- | --- | --- | --- |
|  | **ALA-fed** | | | | **EPA-fed** | | | **DHA-fed** | |
| Fatty Acid | Females | | Males | Females | | Males | Females | | Males |
| 10:0 | 0.050 ± 0.005 | | 0.054 ± 0.004 | 0.064 ± 0.006 | | 0.084 ± 0.008 | ***0.084 ± 0.005*** | | ***0.106 ± 0.008**** |
| 12:0 | 0.648 ± 0.054 | | 0.696 ± 0.050 | 0.526 ± 0.039 | | 0.611 ± 0.054 | 0.492 ± 0.035 | | 0.518 ± 0.035 |
| 14:0 | 1.194 ± 0.070 | | 1.173 ± 0.048 | 1.063 ± 0.061 | | 1.132 ± 0.064 | 0.953 ± 0.056 | | 1.029 ± 0.051 |
| 15:0 | 0.066 ± 0.006 | | 0.064 ± 0.007 | 0.059 ± 0.005 | | 0.069 ± 0.005 | 0.048 ± 0.008 | | 0.055 ± 0.004 |
| 16:0 | 13.166 ± 0.437 | | 12.865 ± 0.319 | 13.217 ± 0.396 | | 13.432 ± 0.461 | 13.518 ± 0.496 | | 13.708 ± 0.551 |
| 17:0 | ***0.107 ± 0.003*** | | ***0.123 ± 0.004**** | 0.110 ± 0.003 | | 0.121 ± 0.005 | 0.108 ± 0.005 | | 0.118 ± 0.005 |
| 18:0 | 16.880 ± 0.360 | | 17.122 ± 0.299 | 16.582 ± 0.394 | | 16.955 ± 0.342 | 16.552 ± 0.455 | | 16.436 ± 0.418 |
| 20:0 | ***0.278 ± 0.008*** | | ***0.353 ± 0.009***** | ***0.274 ± 0.008*** | | ***0.335 ± 0.007***** | ***0.277 ± 0.007*** | | ***0.330 ± 0.008***** |
| 22:0 | 0.188 ± 0.005 | | 0.196 ± 0.005 | 0.189 ± 0.004 | | 0.189 ± 0.005 | 0.194 ± 0.005 | | 0.187 ± 0.004 |
| 23:0 | ***0.042 ± 0.001*** | | ***0.038 ± 0.001**** | ***0.044 ± 0.001*** | | ***0.038 ± 0.001***** | ***0.046 ± 0.002*** | | ***0.037 ± 0.001***** |
| 24:0 | ***0.078 ± 0.002*** | | ***0.070 ± 0.002**** | ***0.075 ± 0.002*** | | ***0.069 ± 0.002**** | ***0.080 ± 0.002*** | | ***0.068 ± 0.001***** |
| SFAs | 32.697 ± 0.573 | | 32.753 ± 0.443 | 32.203 ± 0.563 | | 33.035 ± 0.580 | 32.352 ± 0.676 | | 32.593 ± 0.695 |
| 12:1 | 0.010 ± 0.001 | | 0.011 ± 0.001 | 0.011 ± 0.001 | | 0.012 ± 0.001 | ***0.009 ± 0.001*** | | ***0.012 ± 0.001**** |
| 14:1 | 0.029 ± 0.003 | | 0.032 ± 0.003 | 0.030 ± 0.002 | | 0.031 ± 0.003 | 0.029 ± 0.003 | | 0.025 ± 0.002 |
| 16:1n-7 | 0.979 ± 0.076 | | 1.149 ± 0.074 | 1.051 ± 0.053 | | 1.180 ± 0.063 | 0.938 ± 0.074 | | 1.061 ± 0.062 |
| 16:1n-9 | ***0.124 ± 0.006*** | | ***0.151 ± 0.006**** | ***0.132 ± 0.005*** | | ***0.182 ± 0.011***** | ***0.118 ± 0.006*** | | ***0.176 ± 0.011***** |
| 18:1n-7 | ***2.232 ± 0.056*** | | ***2.460 ± 0.063**** | ***2.380 ± 0.061*** | | ***2.692 ± 0.078**** | ***2.151 ± 0.067*** | | ***2.595 ± 0.088***** |
| 18:1n-9 | 8.250 ± 0.334 | | 8.545 ± 0.244 | ***8.609 ± 0.264*** | | ***9.662 ± 0.371**** | ***7.775 ± 0.276*** | | ***9.316 ± 0.399**** |
| 20:1n-9 | ***0.287 ± 0.008*** | | ***0.432 ± 0.011***** | ***0.309 ± 0.012*** | | ***0.470 ± 0.013***** | ***0.292 ± 0.009*** | | ***0.457 ± 0.016***** |
| 22:1n-9 | ***0.062 ± 0.006*** | | ***0.094 ± 0.007**** | ***0.064 ± 0.006*** | | ***0.093 ± 0.006**** | ***0.063 ± 0.006*** | | ***0.093 ± 0.005***** |
| 24:1n-9 | ***0.030 ± 0.002*** | | ***0.023 ± 0.001**** | ***0.024 ± 0.002*** | | ***0.036 ± 0.003**** | ***0.016 ± 0.002*** | | ***0.046 ± 0.003***** |
| MUFAs | ***12.002 ± 0.348*** | | ***12.897 ± 0.263**** | ***12.609 ± 0.276*** | | ***14.358 ± 0.384***** | ***11.390 ± 0.294*** | | ***13.781 ± 0.414***** |
| 18:2n-6 | ***18.952 ± 0.508*** | | ***22.082 ± 0.473***** | ***18.135 ± 0.607*** | | ***21.362 ± 0.595***** | ***15.862 ± 0.472*** | | ***18.859 ± 0.619***** |
| 18:3n-6 | ***0.056 ± 0.002*** | | ***0.134 ± 0.005***** | ***0.059 ± 0.002*** | | ***0.126 ± 0.005***** | ***0.058 ± 0.003*** | | ***0.123 ± 0.005***** |
| 20:2n-6 | 0.162 ± 0.008 | | 0.152 ± 0.009 | 0.153 ± 0.007 | | 0.151 ± 0.007 | ***0.177 ± 0.008*** | | ***0.153 ± 0.008**** |
| 20:3n-6 | ***0.692 ± 0.011*** | | ***0.984 ± 0.021***** | ***0.679 ± 0.015*** | | ***0.961 ± 0.018***** | ***0.633 ± 0.017*** | | ***0.872 ± 0.023***** |
| 20:4n-6 | ***9.142 ± 0.176*** | | ***10.365 ± 0.182***** | ***8.360 ± 0.186*** | | ***9.096 ± 0.179**** | 6.533 ± 0.162 | | 6.940 ± 0.158 |
| 22:4n-6 | 0.672 ± 0.015 | | 0.701 ± 0.016 | 0.543 ± 0.012 | | 0.579 ± 0.021 | 0.456 ± 0.012 | | 0.470 ± 0.015 |
| 22:5n-6 | ***2.429 ± 0.048*** | | ***2.811 ± 0.070***** | ***1.680 ± 0.054*** | | ***1.900 ± 0.047**** | ***1.178 ± 0.042*** | | ***1.339 ± 0.050**** |
| N-6 PUFA | ***32.105 ± 0.540*** | | ***37.230 ± 0.512***** | ***29.609 ± 0.637*** | | ***34.175 ± 0.624***** | ***24.896 ± 0.502*** | | ***28.755 ± 0.641***** |
| 18:3n-3, ALA | 0.169 ± 0.008 | | 0.186 ± 0.007 | 0.020 ± 0.005 | | 0.011 ± 0.001 | ***0.045 ± 0.009*** | | ***0.011 ± 0.002***** |
| 18:4n-3 | ***0.101 ± 0.007*** | | ***0.045 ± 0.005***** | ***0.092 ± 0.008*** | | ***0.052 ± 0.003***** | ***0.080 ± 0.012*** | | ***0.051 ± 0.004**** |
| 20:3n-3 | 0.008 ± 0.0003 | | 0.007 ± 0.0004 | ***0.003 ± 0.0002*** | | ***0.002 ± 0.0002**** | ***0.003 ± 0.0002*** | | ***0.002 ± 0.0002***** |
| 20:4n-3 | 0.015 ± 0.007 | | 0.107 ± 0.093 | 0.010 ± 0.001 | | 0.012 ± 0.003 | 0.013 ± 0.002 | | 0.016 ± 0.006 |
| 20:5n-3, EPA | ***0.020 ± 0.001*** | | ***0.029 ± 0.001***** | ***0.153 ± 0.008*** | | ***0.208 ± 0.007***** | 0.011 ± 0.001 | | 0.011 ± 0.001 |
| 22:5n-3, DPAn-3 | | ***0.686 ± 0.017*** | ***0.929 ± 0.049***** | ***1.179 ± 0.033*** | | ***1.617 ± 0.060***** | 0.202 ± 0.005 | | 0.200 ± 0.009 |
| 22:6n-3, DHA | ***13.411 ± 0.264*** | | ***10.592 ± 0.293***** | ***15.704 ± 0.306*** | | ***13.667 ± 0.385***** | ***21.935 ± 0.430*** | | ***20.426 ± 0.509**** |
| N-3 PUFA | ***14.409 ± 0.265*** | | ***11.895 ± 0.311***** | ***17.162 ± 0.308*** | | ***15.569 ± 0.389**** | ***22.289 ± 0.430*** | | ***20.717 ± 0.510**** |
| Total PUFA | ***46.514 ± 0.601*** | | ***49.125 ± 0.599**** | ***46.770 ± 0.708*** | | ***49.744 ± 0.735**** | ***47.185 ± 0.661*** | | ***49.472 ± 0.819**** |
| Total FA | ***91.213 ± 0.901*** | | ***94.775 ± 0.791**** | ***91.583 ± 0.946*** | | ***97.137 ± 1.012***** | ***90.927 ± 0.990*** | | ***95.846 ± 1.151**** |

All values are expressed as means (µmol/g) ± SEM (pooled data, n = 28). Statistically significant differences relative to females within each diet group were determined by independent t-test, with ******* denoting a p-value < 0.05, and ******** a p-value < 0.001. ALA – α-linolenic acid; DHA – docosahexaenoic acid; DPAn-3 – docosapentaenoic acid; EPA – eicosapentaenoic acid; FA – fatty acid; MUFA – monounsaturated fatty acid; PUFA – polyunsaturated fatty acid; SFA – saturated fatty acid.

**Supplemental Table 2** – Heart percent weight of fatty acids in total fatty acids of ALA-, EPA- and DHA-fed female and male mice.

|  | Heart Relative Percent (Fatty Acid in % Total Fatty Acids) | | | | | | | | |
| --- | --- | --- | --- | --- | --- | --- | --- | --- | --- |
|  | **ALA-fed** | | | | **EPA-fed** | | | **DHA-fed** | |
| Fatty Acid | Females | | Males | Females | | Males | Females | | Males |
| 10:0 | 0.031 ± 0.002 | | 0.032 ± 0.002 | 0.040 ± 0.003 | | 0.049 ± 0.004 | 0.053 ± 0.003 | | 0.062 ± 0.004 |
| 12:0 | 0.462 ± 0.033 | | 0.485 ± 0.030 | 0.376 ± 0.023 | | 0.409 ± 0.030 | 0.349 ± 0.019 | | 0.352 ± 0.021 |
| 14:0 | 0.978 ± 0.042 | | 0.937 ± 0.028 | 0.869 ± 0.037 | | 0.870 ± 0.035 | 0.776 ± 0.035 | | 0.797 ± 0.028 |
| 15:0 | 0.057 ± 0.004 | | 0.054 ± 0.005 | 0.051 ± 0.004 | | 0.056 ± 0.003 | 0.041 ± 0.006 | | 0.045 ± 0.003 |
| 16:0 | ***12.234 ± 0.184*** | | ***11.566 ± 0.131**** | ***12.206 ± 0.156*** | | ***11.648 ± 0.167**** | 12.456 ± 0.212 | | 11.948 ± 0.207 |
| 17:0 | ***0.105 ± 0.002*** | | ***0.116 ± 0.002**** | 0.107 ± 0.002 | | 0.111 ± 0.002 | 0.105 ± 0.002 | | 0.108 ± 0.003 |
| 18:0 | ***17.522 ± 0.103*** | | ***17.126 ± 0.0898*** | ***17.041 ± 0.142*** | | ***16.440 ± 0.109**** | ***17.007 ± 0.137*** | | ***16.045 ± 0.113***** |
| 20:0 | ***0.316 ± 0.005*** | | ***0.387 ± 0.007***** | ***0.310 ± 0.006*** | | ***0.357 ± 0.005***** | ***0.314 ± 0.003*** | | ***0.355 ± 0.005***** |
| 22:0 | 0.234 ± 0.005 | | 0.235 ± 0.005 | ***0.234 ± 0.004*** | | ***0.220 ± 0.005**** | ***0.240 ± 0.004*** | | ***0.221 ± 0.004**** |
| 23:0 | ***0.055 ± 0.002*** | | ***0.048 ± 0.001**** | ***0.057 ± 0.001*** | | ***0.046 ± 0.001***** | ***0.059 ± 0.001*** | | ***0.046 ± 0.001***** |
| 24:0 | ***0.105 ± 0.003*** | | ***0.091 ± 0.002***** | ***0.101 ± 0.002*** | | ***0.087 ± 0.003***** | ***0.107 ± 0.002*** | | ***0.087 ± 0.002***** |
| SFAs | ***32.101 ± 0.226*** | | ***31.078 ± 0.173***** | ***31.393 ± 0.271*** | | ***30.293 ± 0.230**** | ***31.507 ± 0.332*** | | ***30.066 ± 0.273**** |
| 12:1 | 0.008 ± 0.001 | | 0.007 ± 0.001 | 0.008 ± 0.001 | | 0.008 ± 0.001 | ***0.006 ± 0.001*** | | ***0.008 ± 0.001**** |
| 14:1 | 0.023 ± 0.002 | | 0.025 ± 0.002 | 0.024 ± 0.002 | | 0.023 ± 0.002 | 0.023 ± 0.002 | | 0.019 ± 0.001 |
| 16:1n-7 | 0.889 ± 0.056 | | 1.021 ± 0.057 | 0.962 ± 0.039 | | 1.010 ± 0.039 | 0.849 ± 0.059 | | 0.915 ± 0.040 |
| 16:1n-9 | ***0.114 ± 0.004*** | | ***0.135 ± 0.004***** | ***0.121 ± 0.003*** | | ***0.156 ± 0.007***** | ***0.108 ± 0.004*** | | ***0.152 ± 0.007***** |
| 18:1n-7 | ***2.301 ± 0.032*** | | ***2.448 ± 0.051**** | ***2.431 ± 0.033*** | | ***2.582 ± 0.035**** | ***2.198 ± 0.046*** | | ***2.509 ± 0.043***** |
| 18:1n-9 | 8.445 ± 0.219 | | 8.491 ± 0.189 | 8.795 ± 0.205 | | 9.233 ± 0.207 | ***7.941 ± 0.204*** | | ***8.960 ± 0.204***** |
| 20:1n-9 | ***0.327 ± 0.008*** | | ***0.472 ± 0.010***** | ***0.348 ± 0.012*** | | ***0.498 ± 0.010***** | ***0.331 ± 0.011*** | | ***0.488 ± 0.013***** |
| 22:1n-9 | ***0.075 ± 0.007*** | | ***0.111 ± 0.007***** | ***0.079 ± 0.007*** | | ***0.107 ± 0.006**** | ***0.076 ± 0.006*** | | ***0.108 ± 0.005***** |
| 24:1n-9 | ***0.041 ± 0.003*** | | ***0.030 ± 0.002**** | ***0.032 ± 0.003*** | | ***0.045 ± 0.003**** | ***0.021 ± 0.002*** | | ***0.059 ± 0.003***** |
| MUFAs | 12.221 ± 0.285 | | 12.740 ± 0.275 | ***12.799 ± 0.258*** | | ***13.663 ± 0.260**** | ***11.554 ± 0.282*** | | ***13.219 ± 0.269***** |
| 18:2n-6 | ***19.340 ± 0.207*** | | ***21.786 ± 0.318***** | ***18.349 ± 0.355*** | | ***20.427 ± 0.400***** | ***16.059 ± 0.194*** | | ***18.114 ± 0.325***** |
| 18:3n-6 | ***0.058 ± 0.002*** | | ***0.132 ± 0.004***** | ***0.059 ± 0.002*** | | ***0.119 ± 0.003***** | ***0.058 ± 0.002*** | | ***0.117 ± 0.003***** |
| 20:2n-6 | 0.185 ± 0.010 | | 0.165 ± 0.009 | 0.172 ± 0.008 | | 0.161 ± 0.008 | ***0.198 ± 0.008*** | | ***0.163 ± 0.007**** |
| 20:3n-6 | ***0.779 ± 0.014*** | | ***1.061 ± 0.014***** | ***0.756 ± 0.014*** | | ***1.007 ± 0.015***** | ***0.705 ± 0.016*** | | ***0.919 ± 0.015***** |
| 20:4n-6 | ***10.202 ± 0.155*** | | ***11.107 ± 0.097***** | 9.218 ± 0.123 | | 9.458 ± 0.123 | 7.211 ± 0.100 | | 7.290 ± 0.126 |
| 22:4n-6 | 0.825 ± 0.024 | | 0.822 ± 0.016 | 0.657 ± 0.013 | | 0.658 ± 0.022 | 0.554 ± 0.015 | | 0.541 ± 0.018 |
| 22:5n-6 | ***2.969 ± 0.091*** | | ***3.272 ± 0.068**** | 2.012 ± 0.056 | | 2.162 ± 0.069 | 1.422 ± 0.054 | | 1.539 ± 0.065 |
| N-6 PUFA | ***34.358 ± 0.254*** | | ***38.345 ± 0.349***** | ***31.223 ± 0.309*** | | ***33.993 ± 0.359***** | ***26.208 ± 0.278*** | | ***28.683 ± 0.377***** |
| 18:3n-3, ALA | 0.170 ± 0.006 | | 0.182 ± 0.006 | 0.020 ± 0.005 | | 0.011 ± 0.001 | ***0.045 ± 0.009*** | | ***0.010 ± 0.002***** |
| 18:4n-3 | ***0.101 ± 0.007*** | | ***0.043 ± 0.004***** | ***0.093 ± 0.008*** | | ***0.049 ± 0.002***** | ***0.080 ± 0.011*** | | ***0.050 ± 0.003**** |
| 20:3n-3 | 0.009 ± 0.000 | | 0.008 ± 0.000 | 0.003 ± 0.000 | | 0.003 ± 0.000 | ***0.003 ± 0.000*** | | ***0.002 ± 0.000***** |
| 20:4n-3 | 0.016 ± 0.006 | | 0.104 ± 0.089 | 0.011 ± 0.001 | | 0.012 ± 0.003 | 0.014 ± 0.002 | | 0.017 ± 0.007 |
| 20:5n-3, EPA | ***0.022 ± 0.001*** | | ***0.031 ± 0.001***** | ***0.166 ± 0.007*** | | ***0.217 ± 0.008***** | 0.012 ± 0.001 | | 0.011 ± 0.001 |
| 22:5n-3, DPAn-3 | | ***0.833 ± 0.021*** | ***1.081 ± 0.056***** | ***1.412 ± 0.030*** | | ***1.826 ± 0.062***** | 0.244 ± 0.007 | | 0.227 ± 0.008 |
| 22:6n-3, DHA | ***16.176 ± 0.323*** | | ***12.274 ± 0.335***** | ***18.777 ± 0.393*** | | ***15.344 ± 0.367***** | ***26.188 ± 0.357*** | | ***23.150 ± 0.492***** |
| N-3 PUFA | ***17.439 ± 0.339*** | | ***13.855 ± 0.385***** | ***20.624 ± 0.393*** | | ***17.622 ± 0.415***** | ***26.675 ± 0.361*** | | ***23.547 ± 0.500***** |
| Total PUFA | 51.796 ± 0.441 | | 52.199 ± 0.321 | 51.847 ± 0.261 | | 51.615 ± 0.369 | 52.883 ± 0.409 | | 52.231 ± 0.432 |
| Total FA | 96.119 ± 0.081 | | 96.017 ± 0.128 | ***96.039 ± 0.087*** | | ***95.571 ± 0.110**** | ***95.944 ± 0.100*** | | ***95.515 ± 0.105**** |

All values are expressed as % fatty acid in total heart fatty acids ± SEM (pooled data, n = 28). Statistically significant differences relative to females within each diet group were determined by independent t-test, with ******* denoting a p-value < 0.05, and ******** a p-value < 0.001. ALA – α-linolenic acid; DHA – docosahexaenoic acid; DPAn-3 – docosapentaenoic acid; EPA – eicosapentaenoic acid; FA – fatty acid; MUFA – monounsaturated fatty acid; PUFA – polyunsaturated fatty acid; SFA – saturated fatty acid.

**Supplemental Table** 3 – Perirenal adipose tissue (PRAT) fatty acid concentrations of ALA-, EPA- and DHA-fed female and male mice.

|  | PRAT Concentration (µmol/g) | | | | | | | | |
| --- | --- | --- | --- | --- | --- | --- | --- | --- | --- |
|  | **ALA-fed** | | | | **EPA-fed** | | | **DHA-fed** | |
| Fatty Acid | Females | | Males | Females | | Males | Females | | Males |
| 10:0 | 5.376 ± 0.645 | | 7.061 ± 0.657 | 5.440 ± 0.672 | | 7.043 ± 0.616 | ***5.377 ± 0.647*** | | ***7.647 ± 0.587**** |
| 12:0 | ***244.4 ± 13.9*** | | ***317.4 ± 9.6***** | ***253.7 ± 10.7*** | | ***316.2 ± 10.3***** | ***258.2 ± 11.5*** | | ***323.6 ± 9.3***** |
| 14:0 | ***210.2 ± 8.7*** | | ***242.1 ± 3.6**** | ***222.7 ± 7.0*** | | ***244.3 ± 3.5**** | ***222.8 ± 7.7*** | | ***245.9 ± 4.0**** |
| 15:0 | ***3.297 ± 0.134*** | | ***4.256 ± 0.087***** | ***3.615 ± 0.112*** | | ***4.289 ± 0.079***** | ***3.482 ± 0.108*** | | ***4.212 ± 0.105***** |
| 16:0 | ***792.6 ± 29.2*** | | ***901.9 ± 11.1**** | ***855.5 ± 25.1*** | | ***921.5 ± 10.0**** | ***823.5 ± 26.1*** | | ***927.1 ± 12.5***** |
| 17:0 | ***2.342 ± 0.076*** | | ***2.594 ± 0.055**** | 2.539 ± 0.062 | | 2.539 ± 0.050 | 2.611 ± 0.059 | | 2.679 ± 0.065 |
| 18:0 | ***94.971 ± 3.172*** | | ***84.472 ± 1.337**** | ***97.827 ± 2.365*** | | ***81.256 ± 1.664***** | ***104.461 ± 2.265*** | | ***87.175 ± 1.506***** |
| 20:0 | ***2.763 ± 0.097*** | | ***3.795 ± 0.107***** | ***2.741 ± 0.085*** | | ***3.292 ± 0.128***** | ***3.007 ± 0.112*** | | ***3.502 ± 0.091**** |
| 22:0 | 0.634 ± 0.019 | | 0.683 ± 0.024 | 0.608 ± 0.018 | | 0.595 ± 0.027 | 0.692 ± 0.029 | | 0.633 ± 0.020 |
| 23:0 | ***0.173 ± 0.006*** | | ***0.149 ± 0.006**** | ***0.181 ± 0.005*** | | ***0.144 ± 0.005***** | ***0.189 ± 0.008*** | | ***0.155 ± 0.006***** |
| 24:0 | 0.424 ± 0.012 | | 0.386 ± 0.016 | ***0.409 ± 0.013*** | | ***0.339 ± 0.015***** | ***0.473 ± 0.023*** | | ***0.369 ± 0.013***** |
| SFAs | ***1357.2 ± 33.6*** | | ***1564.8 ± 15.2***** | ***1445.2 ± 28.3*** | | ***1581.5 ± 14.9***** | ***1424.8 ± 29.6*** | | ***1603.0 ± 16.1***** |
| 12:1 | ***3.357 ± 0.219*** | | ***4.776 ± 0.155***** | ***3.671 ± 0.199*** | | ***5.283 ± 0.193***** | ***3.258 ± 0.187*** | | ***4.882 ± 0.134***** |
| 14:1 | ***12.571 ± 0.692*** | | ***15.534 ± 0.305***** | ***13.955 ± 0.681*** | | ***17.187 ± 0.455***** | ***12.070 ± 0.632*** | | ***15.781 ± 0.311***** |
| 16:1n-7 | ***273.8 ± 13.7*** | | ***357.8 ± 8.9***** | ***304.6 ± 13.8*** | | ***385.1 ± 11.9***** | ***264.1 ± 13.8*** | | ***362.3 ± 9.4***** |
| 16:1n-9 | ***12.188 ± 0.555*** | | ***14.770 ± 0.349***** | ***13.232 ± 0.608*** | | ***15.563 ± 0.513**** | ***12.545 ± 0.624*** | | ***14.919 ± 0.513**** |
| 18:1n-7 | ***65.306 ± 2.714*** | | ***79.725 ± 2.067***** | ***72.949 ± 2.631*** | | ***80.827 ± 2.128**** | ***61.832 ± 2.384*** | | ***73.862 ± 2.121***** |
| 18:1n-9 | 798.4 ± 31.5 | | 816.4 ± 11.4 | 859.7 ± 26.8 | | 841.8 ± 12.3 | 841.9 ± 29.7 | | 835.3 ± 14.9 |
| 20:1n-9 | ***14.530 ± 0.533*** | | ***19.503 ± 0.341***** | ***15.277 ± 0.501*** | | ***18.048 ± 0.253***** | ***15.795 ± 0.419*** | | ***18.445 ± 0.434***** |
| 22:1n-9 | ***0.842 ± 0.033*** | | ***1.444 ± 0.051***** | ***0.798 ± 0.055*** | | ***1.285 ± 0.073***** | ***0.885 ± 0.046*** | | ***1.297 ± 0.056***** |
| 24:1n-9 | 0.192 ± 0.012 | | 0.182 ± 0.013 | ***0.166 ± 0.009*** | | ***0.196 ± 0.009**** | ***0.187 ± 0.009*** | | ***0.244 ± 0.016**** |
| MUFAs | ***1181.2 ± 34.5*** | | ***1310.1 ± 14.6**** | ***1284.4 ± 30.3*** | | ***1365.3 ± 17.3**** | ***1212.6 ± 32.8*** | | ***1327.0 ± 17.7**** |
| 18:2n-6 | ***536.4 ± 18.1*** | | ***623.7 ± 12.4***** | ***545.9 ± 14.2*** | | ***602.8 ± 10.9**** | ***571.9 ± 15.1*** | | ***615.7 ± 13.3**** |
| 18:3n-6 | ***1.854 ± 0.067*** | | ***1.625 ± 0.053**** | 1.738 ± 0.054 | | 1.618 ± 0.060 | 1.788 ± 0.069 | | 1.614 ± 0.055 |
| 20:2n-6 | ***2.262 ± 0.078*** | | ***2.595 ± 0.107**** | 2.215 ± 0.070 | | 2.432 ± 0.086 | 2.439 ± 0.098 | | 2.324 ± 0.081 |
| 20:3n-6 | ***3.914 ± 0.109*** | | ***5.266 ± 0.115***** | ***3.961 ± 0.107*** | | ***5.115 ± 0.108***** | ***3.935 ± 0.092*** | | ***5.182 ± 0.101***** |
| 20:4n-6 | ***10.616 ± 0.253*** | | ***9.528 ± 0.184**** | 10.244 ± 0.212 | | 9.866 ± 0.279 | ***10.058 ± 0.202*** | | ***9.460 ± 0.204**** |
| 22:4n-6 | ***1.956 ± 0.093*** | | ***1.660 ± 0.037**** | 1.737 ± 0.102 | | 1.623 ± 0.043 | 1.891 ± 0.209 | | 1.541 ± 0.042 |
| 22:5n-6 | 1.574 ± 0.043 | | 1.546 ± 0.055 | 1.384 ± 0.051 | | 1.360 ± 0.047 | 1.265 ± 0.050 | | 1.366 ± 0.057 |
| N-6 PUFA | ***558.5 ± 18.1*** | | ***645.9 ± 12.4***** | ***567.1 ± 14.2*** | | ***624.8 ± 10.9**** | ***593.3 ± 15.1*** | | ***637.2 ± 13.3**** |
| 18:3n-3, ALA | ***22.826 ± 0.878*** | | ***29.340 ± 0.541***** | ***1.210 ± 0.068*** | | ***1.523 ± 0.089**** | ***1.098 ± 0.065*** | | ***1.311 ± 0.070**** |
| 18:4n-3 | ***0.866 ± 0.040*** | | ***1.123 ± 0.040***** | ***1.113 ± 0.039*** | | ***1.334 ± 0.041***** | ***1.083 ± 0.032*** | | ***1.350 ± 0.038***** |
| 20:3n-3 | ***0.178 ± 0.006*** | | ***0.210 ± 0.005***** | 0.063 ± 0.003 | | 0.065 ± 0.003 | 0.061 ± 0.003 | | 0.063 ± 0.003 |
| 20:4n-3 | ***0.102 ± 0.015*** | | ***0.364 ± 0.033***** | 0.123 ± 0.017 | | 0.106 ± 0.017 | 0.109 ± 0.016 | | 0.097 ± 0.014 |
| 20:5n-3, EPA | 0.375 ± 0.019 | | 0.412 ± 0.012 | ***3.046 ± 0.164*** | | ***3.818 ± 0.141***** | 0.150 ± 0.006 | | 0.151 ± 0.005 |
| 22:5n-3, DPAn-3 | | 0.740 ± 0.027 | 0.813 ± 0.034 | ***1.586 ± 0.047*** | | ***2.068 ± 0.082***** | 0.260 ± 0.011 | | 0.243 ± 0.007 |
| 22:6n-3, DHA | ***3.577 ± 0.112*** | | ***2.520 ± 0.056***** | ***4.865 ± 0.123*** | | ***4.065 ± 0.099***** | 8.828 ± 0.255 | | 9.051 ± 0.217 |
| N-3 PUFA | ***28.7 ± 0.89*** | | ***34.8 ± 0.55***** | ***12.0 ± 0.23*** | | ***13.0 ± 0.22**** | 11.6 ± 0.27 | | 12.3 ± 0.23 |
| Total PUFA | ***587.2 ± 18.1*** | | ***680.7 ± 12.5***** | ***579.2 ± 14.2*** | | ***637.8 ± 10.9**** | ***604.9 ± 15.1*** | | ***649.4 ± 13.3**** |
| Total FA | ***3125.6 ± 51.4*** | | ***3555.6 ± 24.5***** | ***3308.8 ± 43.8*** | | ***3584.5 ± 25.3***** | ***3242.3 ± 46.7*** | | ***3579.4 ± 27.4***** |

All values are expressed as means (µmol/g) ± SEM (pooled data, n = 28). Statistically significant differences relative to females within each diet group were determined by independent t-test, with ******* denoting a p-value < 0.05, and ******** a p-value < 0.001. ALA – α-linolenic acid; DHA – docosahexaenoic acid; DPAn-3 – docosapentaenoic acid; EPA – eicosapentaenoic acid; FA – fatty acid; MUFA – monounsaturated fatty acid; PUFA – polyunsaturated fatty acid; SFA – saturated fatty acid.

**Supplemental Table 4** – Perirenal adipose tissue (PRAT) percent weight of fatty acids in total fatty acids of ALA-, EPA- and DHA-fed female and male mice.

|  | PRAT Relative Percent (Fatty Acid in % Total Fatty Acids) | | | | | | | | |
| --- | --- | --- | --- | --- | --- | --- | --- | --- | --- |
|  | **ALA-fed** | | | | **EPA-fed** | | | **DHA-fed** | |
| Fatty Acid | Females | | Males | Females | | Males | Females | | Males |
| 10:0 | 0.104 ± 0.011 | | 0.128 ± 0.012 | 0.104 ± 0.012 | | 0.127 ± 0.011 | ***0.104 ± 0.011*** | | ***0.139 ± 0.011**** |
| 12:0 | ***5.753 ± 0.164*** | | ***6.728 ± 0.181***** | ***5.732 ± 0.156*** | | ***6.656 ± 0.208***** | ***5.965 ± 0.169*** | | ***6.833 ± 0.184**** |
| 14:0 | 5.736 ± 0.044 | | 5.855 ± 0.063 | 5.759 ± 0.060 | | 5.874 ± 0.082 | 5.876 ± 0.072 | | 5.920 ± 0.081 |
| 15:0 | ***0.096 ± 0.001*** | | ***0.109 ± 0.002***** | ***0.099 ± 0.001*** | | ***0.109 ± 0.002***** | ***0.098 ± 0.001*** | | ***0.107 ± 0.002***** |
| 16:0 | 24.452 ± 0.223 | | 24.511 ± 0.242 | 24.893 ± 0.247 | | 24.867 ± 0.203 | 24.428 ± 0.205 | | 25.060 ± 0.265 |
| 17:0 | 0.077 ± 0.001 | | 0.074 ± 0.001 | ***0.078 ± 0.001*** | | ***0.072 ± 0.001**** | ***0.083 ± 0.002*** | | ***0.076 ± 0.002**** |
| 18:0 | ***3.298 ± 0.083*** | | ***2.550 ± 0.044***** | ***3.201 ± 0.084*** | | ***2.444 ± 0.066***** | ***3.499 ± 0.096*** | | ***2.622 ± 0.056***** |
| 20:0 | ***0.106 ± 0.004*** | | ***0.126 ± 0.004***** | 0.099 ± 0.004 | | 0.109 ± 0.005 | 0.112 ± 0.006 | | 0.116 ± 0.003 |
| 22:0 | 0.027 ± 0.002 | | 0.025 ± 0.001 | 0.024 ± 0.001 | | 0.021 ± 0.001 | ***0.028 ± 0.002*** | | ***0.023 ± 0.001**** |
| 23:0 | ***0.008 ± 0.000*** | | ***0.006 ± 0.000***** | ***0.007 ± 0.000*** | | ***0.005 ± 0.000***** | ***0.008 ± 0.000*** | | ***0.006 ± 0.000***** |
| 24:0 | ***0.020 ± 0.001*** | | ***0.015 ± 0.001**** | ***0.018 ± 0.001*** | | ***0.013 ± 0.001***** | ***0.021 ± 0.002*** | | ***0.014 ± 0.001***** |
| SFAs | 39.675 ± 0.283 | | 40.127 ± 0.305 | 40.014 ± 0.380 | | 40.298 ± 0.348 | 40.221 ± 0.335 | | 40.917 ± 0.345 |
| 12:1 | ***0.078 ± 0.003*** | | ***0.100 ± 0.003***** | ***0.081 ± 0.003*** | | ***0.110 ± 0.003***** | ***0.073 ± 0.003*** | | ***0.102 ± 0.003***** |
| 14:1 | ***0.336 ± 0.010*** | | ***0.372 ± 0.006**** | ***0.354 ± 0.011*** | | ***0.408 ± 0.008***** | ***0.311 ± 0.010*** | | ***0.376 ± 0.006***** |
| 16:1n-7 | ***8.276 ± 0.221*** | | ***9.637 ± 0.214***** | ***8.701 ± 0.237*** | | ***10.275 ± 0.255***** | ***7.653 ± 0.234*** | | ***9.698 ± 0.203***** |
| 16:1n-9 | ***0.371 ± 0.007*** | | ***0.397 ± 0.006**** | ***0.380 ± 0.012*** | | ***0.415 ± 0.011**** | ***0.366 ± 0.010*** | | ***0.398 ± 0.011**** |
| 18:1n-7 | ***2.209 ± 0.030*** | | ***2.380 ± 0.048**** | 2.330 ± 0.041 | | 2.397 ± 0.051 | ***2.021 ± 0.043*** | | ***2.193 ± 0.050**** |
| 18:1n-9 | ***27.092 ± 0.256*** | | ***24.410 ± 0.151***** | ***27.564 ± 0.376*** | | ***25.004 ± 0.255***** | ***27.502 ± 0.371*** | | ***24.824 ± 0.243***** |
| 20:1n-9 | ***0.548 ± 0.014*** | | ***0.642 ± 0.012***** | ***0.544 ± 0.017*** | | ***0.590 ± 0.008**** | 0.581 ± 0.023 | | 0.603 ± 0.012 |
| 22:1n-9 | ***0.037 ± 0.003*** | | ***0.052 ± 0.002***** | ***0.032 ± 0.002*** | | ***0.046 ± 0.003***** | ***0.037 ± 0.003*** | | ***0.046 ± 0.002**** |
| 24:1n-9 | 0.009 ± 0.001 | | 0.007 ± 0.001 | 0.007 ± 0.000 | | 0.008 ± 0.000 | 0.008 ± 0.001 | | 0.010 ± 0.001 |
| MUFAs | 38.956 ± 0.326 | | 37.998 ± 0.359 | 39.993 ± 0.423 | | 39.252 ± 0.469 | 38.551 ± 0.445 | | 38.250 ± 0.408 |
| 18:2n-6 | 18.193 ± 0.217 | | 18.520 ± 0.314 | 17.442 ± 0.227 | | 17.787 ± 0.295 | 18.692 ± 0.259 | | 18.181 ± 0.312 |
| 18:3n-6 | ***0.062 ± 0.001*** | | ***0.048 ± 0.001***** | ***0.055 ± 0.001*** | | ***0.047 ± 0.002***** | ***0.058 ± 0.002*** | | ***0.047 ± 0.001***** |
| 20:2n-6 | 0.086 ± 0.004 | | 0.085 ± 0.004 | 0.080 ± 0.004 | | 0.079 ± 0.003 | ***0.090 ± 0.005*** | | ***0.076 ± 0.003**** |
| 20:3n-6 | ***0.147 ± 0.003*** | | ***0.171 ± 0.003***** | ***0.139 ± 0.003*** | | ***0.165 ± 0.003***** | ***0.142 ± 0.004*** | | ***0.167 ± 0.003***** |
| 20:4n-6 | ***0.403 ± 0.016*** | | ***0.307 ± 0.005***** | ***0.362 ± 0.013*** | | ***0.316 ± 0.008**** | ***0.364 ± 0.014*** | | ***0.304 ± 0.006***** |
| 22:4n-6 | ***0.085 ± 0.007*** | | ***0.058 ± 0.001**** | 0.071 ± 0.008 | | 0.057 ± 0.001 | ***0.080 ± 0.012*** | | ***0.054 ± 0.001**** |
| 22:5n-6 | ***0.065 ± 0.003*** | | ***0.054 ± 0.002**** | 0.053 ± 0.002 | | 0.047 ± 0.002 | 0.051 ± 0.003 | | 0.048 ± 0.002 |
| N-6 PUFA | 19.040 ± 0.233 | | 19.243 ± 0.319 | 18.201 ± 0.239 | | 18.498 ± 0.295 | 19.476 ± 0.275 | | 18.877 ± 0.313 |
| 18:3n-3, ALA | ***0.762 ± 0.007*** | | ***0.865 ± 0.012***** | 0.038 ± 0.002 | | 0.045 ± 0.003 | 0.036 ± 0.002 | | 0.039 ± 0.002 |
| 18:4n-3 | ***0.029 ± 0.001*** | | ***0.033 ± 0.001**** | ***0.035 ± 0.001*** | | ***0.039 ± 0.001**** | ***0.035 ± 0.001*** | | ***0.039 ± 0.001**** |
| 20:3n-3 | 0.007 ± 0.000 | | 0.007 ± 0.000 | 0.002 ± 0.000 | | 0.002 ± 0.000 | 0.002 ± 0.000 | | 0.002 ± 0.000 |
| 20:4n-3 | ***0.004 ± 0.001*** | | ***0.012 ± 0.001***** | 0.004 ± 0.001 | | 0.003 ± 0.001 | 0.004 ± 0.001 | | 0.003 ± 0.000 |
| 20:5n-3, EPA | 0.014 ± 0.000 | | 0.013 ± 0.000 | ***0.104 ± 0.004*** | | ***0.122 ± 0.005**** | 0.005 ± 0.000 | | 0.005 ± 0.000 |
| 22:5n-3, DPAn-3 | | 0.031 ± 0.002 | 0.028 ± 0.001 | ***0.061 ± 0.003*** | | ***0.072 ± 0.003**** | ***0.010 ± 0.001*** | | ***0.008 ± 0.000**** |
| 22:6n-3, DHA | ***0.147 ± 0.007*** | | ***0.088 ± 0.002***** | ***0.186 ± 0.008*** | | ***0.141 ± 0.004***** | 0.344 ± 0.014 | | 0.314 ± 0.008 |
| N-3 PUFA | ***0.992 ± 0.012*** | | ***1.045 ± 0.013**** | 0.430 ± 0.013 | | 0.423 ± 0.009 | 0.437 ± 0.017 | | 0.411 ± 0.010 |
| Total PUFA | 20.033 ± 0.238 | | 20.289 ± 0.325 | 18.631 ± 0.242 | | 18.921 ± 0.298 | 19.913 ± 0.284 | | 19.288 ± 0.314 |
| Total FA | ***98.663 ± 0.02*** | | ***98.414 ± 0.02***** | ***98.639 ± 0.02*** | | ***98.471 ± 0.03***** | ***98.686 ± 0.03*** | | ***98.455 ± 0.02***** |

All values are expressed as % fatty acid in total PRAT fatty acids ± SEM (pooled data, n = 28). Statistically significant differences relative to females within each diet group were determined by independent t-test, with ******* denoting a p-value < 0.05, and ******** a p-value < 0.001. ALA – α-linolenic acid; DHA – docosahexaenoic acid; DPAn-3 – docosapentaenoic acid; EPA – eicosapentaenoic acid; FA – fatty acid; MUFA – monounsaturated fatty acid; PUFA – polyunsaturated fatty acid; SFA – saturated fatty acid.

**Supplemental Table** 5 – Brain fatty acid concentrations of ALA-, EPA- and DHA-fed female and male mice.

|  | Brain Concentration (µmol/g) | | | | | | | | |
| --- | --- | --- | --- | --- | --- | --- | --- | --- | --- |
|  | **ALA-fed** | | | | **EPA-fed** | | | **DHA-fed** | |
| Fatty Acid | Females | | Males | Females | | Males | Females | | Males |
| 10:0 | 0.035 ± 0.003 | | 0.037 ± 0.004 | 0.056 ± 0.003 | | 0.056 ± 0.004 | 0.064 ± 0.007 | | 0.082 ± 0.008 |
| 12:0 | 0.025 ± 0.002 | | 0.029 ± 0.003 | 0.023 ± 0.003 | | 0.031 ± 0.004 | ***0.022 ± 0.002*** | | ***0.034 ± 0.005**** |
| 14:0 | 0.509 ± 0.024 | | 0.476 ± 0.028 | 0.503 ± 0.028 | | 0.478 ± 0.026 | 0.468 ± 0.028 | | 0.471 ± 0.027 |
| 15:0 | 0.088 ± 0.004 | | 0.086 ± 0.005 | 0.088 ± 0.004 | | 0.087 ± 0.004 | 0.081 ± 0.004 | | 0.083 ± 0.004 |
| 16:0 | 35.732 ± 0.988 | | 34.459 ± 1.320 | 35.240 ± 1.021 | | 34.403 ± 1.079 | 33.175 ± 1.135 | | 33.117 ± 1.120 |
| 17:0 | 0.268 ± 0.008 | | 0.268 ± 0.010 | 0.270 ± 0.008 | | 0.265 ± 0.008 | 0.257 ± 0.008 | | 0.258 ± 0.009 |
| 18:0 | 32.841 ± 0.586 | | 32.059 ± 0.756 | 32.856 ± 0.658 | | 31.827 ± 0.648 | 31.899 ± 0.713 | | 31.178 ± 0.725 |
| 20:0 | 0.642 ± 0.013 | | 0.645 ± 0.015 | 0.628 ± 0.013 | | 0.630 ± 0.014 | 0.636 ± 0.019 | | 0.619 ± 0.017 |
| 22:0 | 0.704 ± 0.012 | | 0.726 ± 0.014 | 0.704 ± 0.016 | | 0.722 ± 0.013 | 0.706 ± 0.014 | | 0.727 ± 0.017 |
| 23:0 | 0.190 ± 0.004 | | 0.199 ± 0.004 | 0.196 ± 0.006 | | 0.204 ± 0.005 | 0.199 ± 0.006 | | 0.210 ± 0.005 |
| 24:0 | 1.066 ± 0.020 | | 1.085 ± 0.020 | 1.070 ± 0.028 | | 1.096 ± 0.023 | 1.078 ± 0.021 | | 1.131 ± 0.025 |
| SFAs | 72.099 ± 1.662 | | 70.068 ± 2.179 | 71.635 ± 1.788 | | 69.801 ± 1.828 | 68.585 ± 1.957 | | 67.911 ± 1.963 |
| 12:1 | 0.006 ± 0.000 | | 0.006 ± 0.001 | 0.008 ± 0.000 | | 0.008 ± 0.001 | 0.010 ± 0.001 | | 0.012 ± 0.001 |
| 14:1 | 0.048 ± 0.005 | | 0.037 ± 0.006 | 0.056 ± 0.006 | | 0.042 ± 0.006 | 0.057 ± 0.008 | | 0.053 ± 0.006 |
| 16:1n-7 | 0.941 ± 0.025 | | 0.993 ± 0.045 | 0.943 ± 0.028 | | 0.977 ± 0.032 | 0.846 ± 0.026 | | 0.915 ± 0.028 |
| 16:1n-9 | 0.380 ± 0.016 | | 0.369 ± 0.019 | 0.383 ± 0.018 | | 0.374 ± 0.015 | 0.363 ± 0.019 | | 0.372 ± 0.017 |
| 18:1n-7 | 6.029 ± 0.116 | | 6.052 ± 0.153 | 6.035 ± 0.128 | | 5.965 ± 0.127 | 5.733 ± 0.122 | | 5.774 ± 0.147 |
| 18:1n-9 | 24.938 ± 0.456 | | 24.915 ± 0.721 | 25.085 ± 0.516 | | 24.616 ± 0.504 | 24.067 ± 0.507 | | 24.162 ± 0.599 |
| 20:1n-9 | 2.545 ± 0.041 | | 2.503 ± 0.050 | 2.496 ± 0.086 | | 2.468 ± 0.042 | 2.523 ± 0.057 | | 2.448 ± 0.066 |
| 22:1n-9 | ***0.277 ± 0.007*** | | ***0.300 ± 0.008**** | 0.265 ± 0.005 | | 0.281 ± 0.007 | ***0.271 ± 0.006*** | | ***0.292 ± 0.007**** |
| 24:1n-9 | 2.119 ± 0.029 | | 2.111 ± 0.033 | 2.129 ± 0.048 | | 2.123 ± 0.034 | 2.183 ± 0.051 | | 2.172 ± 0.044 |
| MUFAs | 37.282 ± 0.697 | | 37.285 ± 1.035 | 37.400 ± 0.837 | | 36.855 ± 0.766 | 36.052 ± 0.798 | | 36.200 ± 0.915 |
| 18:2n-6 | 0.851 ± 0.023 | | 0.888 ± 0.067 | 0.811 ± 0.020 | | 0.782 ± 0.026 | 0.819 ± 0.023 | | 0.784 ± 0.026 |
| 18:3n-6 | 0.073 ± 0.001 | | 0.074 ± 0.002 | 0.073 ± 0.002 | | 0.074 ± 0.001 | 0.072 ± 0.002 | | 0.074 ± 0.001 |
| 20:2n-6 | 0.230 ± 0.007 | | 0.224 ± 0.009 | 0.216 ± 0.006 | | 0.204 ± 0.008 | 0.202 ± 0.008 | | 0.203 ± 0.008 |
| 20:3n-6 | ***0.593 ± 0.013*** | | ***0.634 ± 0.015**** | ***0.597 ± 0.013*** | | ***0.637 ± 0.014**** | ***0.613 ± 0.011*** | | ***0.658 ± 0.014**** |
| 20:4n-6 | 13.825 ± 0.202 | | 13.800 ± 0.242 | 13.788 ± 0.214 | | 13.571 ± 0.183 | 13.320 ± 0.177 | | 13.114 ± 0.193 |
| 22:4n-6 | 3.373 ± 0.030 | | 3.362 ± 0.041 | 3.206 ± 0.124 | | 3.267 ± 0.024 | 3.231 ± 0.032 | | 3.167 ± 0.026 |
| 22:5n-6 | ***0.601 ± 0.012*** | | ***0.697 ± 0.014***** | ***0.477 ± 0.011*** | | ***0.542 ± 0.013***** | ***0.440 ± 0.013*** | | ***0.491 ± 0.017**** |
| N-6 PUFA | 19.546 ± 0.288 | | 19.679 ± 0.389 | 19.170 ± 0.390 | | 19.077 ± 0.270 | 18.697 ± 0.266 | | 18.491 ± 0.287 |
| 18:3n-3, ALA | 0.047 ± 0.006 | | 0.048 ± 0.007 | 0.024 ± 0.004 | | 0.036 ± 0.006 | 0.029 ± 0.007 | | 0.024 ± 0.005 |
| 18:4n-3 | 0.079 ± 0.007 | | 0.067 ± 0.006 | ***0.096 ± 0.008*** | | ***0.068 ± 0.008**** | 0.241 ± 0.153 | | 0.102 ± 0.006 |
| 20:3n-3 | 0.013 ± 0.001 | | 0.012 ± 0.001 | 0.010 ± 0.000 | | 0.010 ± 0.000 | 0.013 ± 0.002 | | 0.011 ± 0.001 |
| 20:4n-3 | 0.018 ± 0.002 | | 0.016 ± 0.001 | 0.021 ± 0.003 | | 0.016 ± 0.002 | 0.035 ± 0.019 | | 0.023 ± 0.008 |
| 20:5n-3, EPA | 0.036 ± 0.002 | | 0.034 ± 0.001 | 0.046 ± 0.001 | | 0.046 ± 0.002 | 0.045 ± 0.001 | | 0.047 ± 0.002 |
| 22:5n-3, DPAn-3 | | ***0.161 ± 0.002*** | ***0.169 ± 0.003**** | ***0.197 ± 0.003*** | | ***0.207 ± 0.002**** | 0.155 ± 0.004 | | 0.152 ± 0.002 |
| 22:6n-3, DHA | 19.042 ± 0.182 | | 18.823 ± 0.224 | 19.475 ± 0.204 | | 19.236 ± 0.157 | 19.863 ± 0.164 | | 19.644 ± 0.127 |
| N-3 PUFA | 19.396 ± 0.202 | | 19.170 ± 0.243 | 19.869 ± 0.223 | | 19.619 ± 0.177 | 20.381 ± 0.350 | | 20.003 ± 0.151 |
| Total PUFA | 38.942 ± 0.490 | | 38.849 ± 0.632 | 39.039 ± 0.613 | | 38.696 ± 0.447 | 39.079 ± 0.616 | | 38.494 ± 0.438 |
| Total FA | 148.324 ± 2.850 | | 146.202 ± 3.847 | 148.073 ± 3.237 | | 145.351 ± 3.041 | 143.716 ± 3.371 | | 142.605 ± 3.316 |

All values are expressed as means (µmol/g) ± SEM (pooled data, n = 28). Statistically significant differences relative to females within each diet group were determined by independent t-test, with ******* denoting a p-value < 0.05, and ******** a p-value < 0.001. ALA – α-linolenic acid; DHA – docosahexaenoic acid; DPAn-3 – docosapentaenoic acid; EPA – eicosapentaenoic acid; FA – fatty acid; MUFA – monounsaturated fatty acid; PUFA – polyunsaturated fatty acid; SFA – saturated fatty acid.

**Supplemental Table 6** – Brain percent weight of fatty acids in total fatty acids of ALA-, EPA- and DHA-fed female and male mice.

|  | Brain Relative Percent (Fatty Acid in % Total Fatty Acids) | | | | | | | | |
| --- | --- | --- | --- | --- | --- | --- | --- | --- | --- |
|  | **ALA-fed** | | | | **EPA-fed** | | | **DHA-fed** | |
| Fatty Acid | Females | | Males | Females | | Males | Females | | Males |
| 10:0 | 0.013 ± 0.001 | | 0.014 ± 0.001 | 0.020 ± 0.001 | | 0.021 ± 0.001 | 0.023 ± 0.002 | | 0.030 ± 0.003 |
| 12:0 | 0.010 ± 0.001 | | 0.012 ± 0.001 | 0.009 ± 0.001 | | 0.013 ± 0.002 | ***0.009 ± 0.001*** | | ***0.014 ± 0.002**** |
| 14:0 | 0.243 ± 0.008 | | 0.229 ± 0.008 | 0.237 ± 0.009 | | 0.231 ± 0.009 | 0.227 ± 0.009 | | 0.229 ± 0.008 |
| 15:0 | 0.044 ± 0.001 | | 0.044 ± 0.001 | 0.044 ± 0.001 | | 0.045 ± 0.002 | 0.042 ± 0.001 | | 0.043 ± 0.001 |
| 16:0 | 19.297 ± 0.252 | | 18.842 ± 0.270 | 18.865 ± 0.204 | | 18.866 ± 0.272 | 18.249 ± 0.259 | | 18.257 ± 0.238 |
| 17:0 | 0.153 ± 0.002 | | 0.155 ± 0.002 | 0.152 ± 0.002 | | 0.153 ± 0.002 | 0.150 ± 0.002 | | 0.150 ± 0.003 |
| 18:0 | 19.770 ± 0.135 | | 19.623 ± 0.114 | 19.602 ± 0.071 | | 19.474 ± 0.114 | ***19.595 ± 0.073*** | | ***19.187 ± 0.082***** |
| 20:0 | 0.425 ± 0.006 | | 0.436 ± 0.009 | 0.413 ± 0.006 | | 0.425 ± 0.008 | 0.429 ± 0.008 | | 0.418 ± 0.006 |
| 22:0 | 0.509 ± 0.008 | | 0.535 ± 0.011 | ***0.504 ± 0.009*** | | ***0.532 ± 0.011**** | 0.522 ± 0.010 | | 0.537 ± 0.007 |
| 23:0 | 0.144 ± 0.004 | | 0.153 ± 0.004 | 0.147 ± 0.004 | | 0.157 ± 0.005 | 0.154 ± 0.005 | | 0.163 ± 0.005 |
| 24:0 | 0.836 ± 0.019 | | 0.868 ± 0.018 | 0.830 ± 0.018 | | 0.876 ± 0.022 | 0.865 ± 0.018 | | 0.906 ± 0.016 |
| SFAs | 41.444 ± 0.337 | | 40.910 ± 0.313 | 40.822 ± 0.221 | | 40.794 ± 0.347 | 40.264 ± 0.279 | | 39.933 ± 0.283 |
| 12:1 | 0.002 ± 0.000 | | 0.003 ± 0.000 | 0.003 ± 0.000 | | 0.003 ± 0.000 | 0.004 ± 0.000 | | 0.005 ± 0.000 |
| 14:1 | 0.022 ± 0.002 | | 0.017 ± 0.003 | 0.025 ± 0.002 | | 0.020 ± 0.002 | 0.027 ± 0.004 | | 0.026 ± 0.003 |
| 16:1n-7 | ***0.505 ± 0.008*** | | ***0.538 ± 0.012**** | ***0.501 ± 0.008*** | | ***0.532 ± 0.009**** | ***0.463 ± 0.006*** | | ***0.502 ± 0.007***** |
| 16:1n-9 | 0.203 ± 0.006 | | 0.199 ± 0.006 | 0.202 ± 0.006 | | 0.203 ± 0.005 | 0.197 ± 0.008 | | 0.203 ± 0.006 |
| 18:1n-7 | ***3.600 ± 0.020*** | | ***3.674 ± 0.022**** | 3.573 ± 0.017 | | 3.623 ± 0.021 | 3.500 ± 0.021 | | 3.524 ± 0.023 |
| 18:1n-9 | 14.896 ± 0.056 | | 15.099 ± 0.112 | 14.858 ± 0.083 | | 14.954 ± 0.080 | 14.698 ± 0.116 | | 14.754 ± 0.103 |
| 20:1n-9 | 1.679 ± 0.028 | | 1.681 ± 0.029 | 1.625 ± 0.050 | | 1.657 ± 0.027 | 1.702 ± 0.039 | | 1.647 ± 0.031 |
| 22:1n-9 | ***0.200 ± 0.005*** | | ***0.222 ± 0.009**** | ***0.189 ± 0.005*** | | ***0.207 ± 0.0068*** | 0.201 ± 0.006 | | 0.215 ± 0.006 |
| 24:1n-9 | 1.657 ± 0.037 | | 1.684 ± 0.039 | 1.647 ± 0.041 | | 1.693 ± 0.044 | 1.748 ± 0.052 | | 1.738 ± 0.044 |
| MUFAs | ***22.764 ± 0.089*** | | ***23.117 ± 0.149**** | 22.625 ± 0.122 | | 22.891 ± 0.122 | 22.540 ± 0.197 | | 22.614 ± 0.166 |
| 18:2n-6 | 0.505 ± 0.010 | | 0.525 ± 0.026 | 0.478 ± 0.008 | | 0.470 ± 0.011 | 0.497 ± 0.010 | | 0.475 ± 0.010 |
| 18:3n-6 | 0.043 ± 0.001 | | 0.044 ± 0.001 | ***0.043 ± 0.001*** | | ***0.045 ± 0.001**** | 0.044 ± 0.001 | | 0.045 ± 0.001 |
| 20:2n-6 | 0.150 ± 0.004 | | 0.149 ± 0.005 | 0.140 ± 0.004 | | 0.135 ± 0.004 | 0.135 ± 0.005 | | 0.135 ± 0.004 |
| 20:3n-6 | ***0.385 ± 0.007*** | | ***0.419 ± 0.007***** | ***0.385 ± 0.006*** | | ***0.421 ± 0.007***** | ***0.408 ± 0.007*** | | ***0.437 ± 0.005***** |
| 20:4n-6 | 8.925 ± 0.073 | | 9.075 ± 0.075 | 8.832 ± 0.076 | | 8.924 ± 0.080 | 8.811 ± 0.101 | | 8.687 ± 0.088 |
| 22:4n-6 | 2.389 ± 0.038 | | 2.428 ± 0.042 | 2.243 ± 0.089 | | 2.359 ± 0.044 | 2.346 ± 0.049 | | 2.303 ± 0.042 |
| 22:5n-6 | ***0.421 ± 0.008*** | | ***0.500 ± 0.011***** | ***0.332 ± 0.007*** | | ***0.387 ± 0.010***** | ***0.317 ± 0.011*** | | ***0.354 ± 0.014**** |
| N-6 PUFA | 12.818 ± 0.117 | | 13.141 ± 0.118 | 12.452 ± 0.132 | | 12.741 ± 0.127 | 12.558 ± 0.159 | | 12.436 ± 0.137 |
| 18:3n-3, ALA | 0.029 ± 0.004 | | 0.029 ± 0.004 | 0.015 ± 0.003 | | 0.022 ± 0.004 | 0.017 ± 0.004 | | 0.015 ± 0.003 |
| 18:4n-3 | 0.045 ± 0.004 | | 0.040 ± 0.004 | ***0.054 ± 0.004*** | | ***0.041 ± 0.005**** | 0.122 ± 0.071 | | 0.060 ± 0.003 |
| 20:3n-3 | 0.009 ± 0.000 | | 0.008 ± 0.000 | 0.006 ± 0.000 | | 0.007 ± 0.000 | 0.009 ± 0.001 | | 0.008 ± 0.001 |
| 20:4n-3 | 0.012 ± 0.002 | | 0.011 ± 0.001 | 0.014 ± 0.002 | | 0.011 ± 0.001 | 0.020 ± 0.009 | | 0.015 ± 0.005 |
| 20:5n-3, EPA | 0.024 ± 0.002 | | 0.022 ± 0.001 | 0.029 ± 0.000 | | 0.030 ± 0.001 | 0.029 ± 0.001 | | 0.031 ± 0.001 |
| 22:5n-3, DPAn-3 | | ***0.113 ± 0.002*** | ***0.121 ± 0.002**** | ***0.137 ± 0.002*** | | ***0.149 ± 0.003***** | 0.111 ± 0.002 | | 0.110 ± 0.002 |
| 22:6n-3, DHA | 13.317 ± 0.216 | | 13.419 ± 0.219 | 13.500 ± 0.189 | | 13.703 ± 0.234 | 14.251 ± 0.296 | | 14.113 ± 0.260 |
| N-3 PUFA | 13.548 ± 0.225 | | 13.649 ± 0.231 | 13.755 ± 0.184 | | 13.961 ± 0.234 | 14.560 ± 0.286 | | 14.352 ± 0.272 |
| Total PUFA | 26.366 ± 0.330 | | 26.791 ± 0.340 | 26.207 ± 0.287 | | 26.702 ± 0.354 | 27.118 ± 0.433 | | 26.788 ± 0.398 |
| Total FA | 90.574 ± 0.362 | | 90.817 ± 0.346 | 89.655 ± 0.218 | | 90.388 ± 0.337 | 89.921 ± 0.431 | | 89.336 ± 0.272 |

All values are expressed as % fatty acid in total brain fatty acids ± SEM (pooled data, n = 28). Statistically significant differences relative to females within each diet group were determined by independent t-test, with ******* denoting a p-value < 0.05, and ******** a p-value < 0.001. ALA – α-linolenic acid; DHA – docosahexaenoic acid; DPAn-3 – docosapentaenoic acid; EPA – eicosapentaenoic acid; FA – fatty acid; MUFA – monounsaturated fatty acid; PUFA – polyunsaturated fatty acid; SFA – saturated fatty acid.

**Supplemental Table 7** – Red blood cell (RBC) fatty acid concentrations of ALA-, EPA- and DHA-fed female and male mice.

|  | RBC Concentration (nmol/g) | | | | | | | | |
| --- | --- | --- | --- | --- | --- | --- | --- | --- | --- |
|  | **ALA-fed** | | | | **EPA-fed** | | | **DHA-fed** | |
| Fatty Acid | Females | | Males | Females | | Males | Females | | Males |
| 12:0 | 11.7 ± 1.62 | | 13.1 ± 2.32 | 6.77 ± 0.78 | | 5.90 ± 0.65 | ***7.03 ± 0.67*** | | ***5.26 ± 0.51**** |
| 14:0 | 62.4 ± 3.46 | | 65.6 ± 3.72 | 54.7 ± 3.97 | | 56.5 ± 4.43 | 56.3 ± 4.75 | | 49.7 ± 5.12 |
| 15:0 | ***6.49 ± 0.29*** | | ***7.71 ± 0.48**** | 6.29 ± 0.42 | | 7.09 ± 0.39 | 6.88 ± 0.36 | | 9.32 ± 2.53 |
| 16:0 | 2541 ± 78.7 | | 2746 ± 87.7 | 2407 ± 106.1 | | 2666 ± 172.7 | 2554 ± 96.1 | | 2592 ± 118.1 |
| 17:0 | ***17.8 ± 0.43*** | | ***20.8 ± 0.79**** | 17.8 ± 0.65 | | 19.5 ± 1.20 | 19.3 ± 0.64 | | 19.1 ± 0.88 |
| 18:0 | 1551 ± 38.6 | | 1448 ± 34.7 | 1421 ± 40.3 | | 1432 ± 85.8 | ***1529 ± 36.1*** | | ***1356 ± 40.7**** |
| 20:0 | ***18.5 ± 0.44*** | | ***25.9 ± 0.83***** | ***18.6 ± 0.68*** | | ***23.5 ± 1.38**** | ***18.5 ± 0.44*** | | ***24.1 ± 0.78***** |
| 22:0 | 55.7 ± 1.57 | | 53.4 ± 1.28 | 57.8 ± 0.83 | | 59.9 ± 3.78 | 60.4 ± 1.81 | | 60.7 ± 2.72 |
| 23:0 | ***6.59 ± 0.23*** | | ***7.52 ± 0.28**** | 7.65 ± 0.28 | | 8.19 ± 0.52 | ***7.22 ± 0.24*** | | ***8.95 ± 0.31***** |
| 24:0 | 79.8 ± 3.95 | | 81.9 ± 3.18 | 87.3 ± 3.13 | | 90.8 ± 7.16 | 90.3 ± 3.54 | | 91.7 ± 4.06 |
| SFAs | 4351 ± 87.8 | | 4470 ± 94.5 | 4085 ± 113.6 | | 4369 ± 193.1 | 4349 ± 102.9 | | 4216 ± 125.1 |
| 14:1 | 1.16 ± 0.20 | | 0.79 ± 0.08 | 0.62 ± 0.08 | | 1.06 ± 0.36 | 0.59 ± 0.08 | | 0.73 ± 0.12 |
| 16:1n-7 | ***65.7 ± 2.87*** | | ***93.7 ± 5.30***** | ***74.5 ± 4.83*** | | ***94.5 ± 6.49**** | ***59.3 ± 4.02*** | | ***83.8 ± 5.01***** |
| 16:1n-9 | ***9.16 ± 0.39*** | | ***11.3 ± 0.62**** | 11.5 ± 1.44 | | 13.2 ± 1.11 | ***8.47 ± 0.44*** | | ***11.3 ± 0.60***** |
| 18:1n-7 | ***162 ± 4.60*** | | ***204 ± 5.59***** | 172 ± 7.13 | | 202 ± 14.45 | ***153 ± 6.05*** | | ***182 ± 6.80**** |
| 18:1n-9 | ***950 ± 24.3*** | | ***1060 ± 25.5**** | 943 ± 34.4 | | 1050 ± 63.8 | 918 ± 27.4 | | 974 ± 31.0 |
| 20:1n-9 | ***24.4 ± 0.43*** | | ***34.3 ± 0.78***** | ***22.8 ± 1.30*** | | ***29.4 ± 1.58**** | ***23.1 ± 0.62*** | | ***29.3 ± 0.84***** |
| 22:1n-9 | 12.5 ± 1.38 | | 15.2 ± 1.72 | 11.0 ± 0.49 | | 14.4 ± 1.75 | ***10.1 ± 0.48*** | | ***11.9 ± 0.73**** |
| 24:1n-9 | 56.2 ± 1.88 | | 54.6 ± 1.70 | 62.1 ± 1.27 | | 64.1 ± 4.14 | 61.7 ± 1.71 | | 67.4 ± 2.30 |
| MUFAs | ***1281 ± 25.0*** | | ***1474 ± 26.8***** | ***1297 ± 35.5*** | | ***1469 ± 66.0**** | ***1234 ± 28.4*** | | ***1360 ± 32.3**** |
| 18:2n-6 | ***1119 ± 31.9*** | | ***1248 ± 33.3**** | 1014 ± 39.0 | | 1126 ± 58.9 | 1119 ± 31.9 | | 1118 ± 42.8 |
| 18:3n-6 | ***7.46 ± 0.26*** | | ***9.06 ± 0.48**** | ***6.43 ± 0.39*** | | ***7.93 ± 0.40**** | ***6.19 ± 0.26*** | | ***8.01 ± 0.47**** |
| 20:2n-6 | ***22.0 ± 1.16*** | | ***25.5 ± 0.96**** | 21.9 ± 1.44 | | 22.4 ± 1.01 | 22.8 ± 1.20 | | 22.5 ± 1.09 |
| 20:3n-6 | ***143 ± 3.03*** | | ***197 ± 3.85***** | ***135 ± 5.21*** | | ***177 ± 11.69**** | ***135 ± 4.49*** | | ***179 ± 4.94***** |
| 20:4n-6 | 2152 ± 43.6 | | 2088 ± 40.6 | 1948 ± 48.4 | | 1980 ± 115.2 | ***2072 ± 44.8*** | | ***1932 ± 52.0**** |
| 22:4n-6 | 219 ± 2.86 | | 223 ± 4.07 | 187 ± 3.94 | | 194 ± 9.77 | 180 ± 3.24 | | 176 ± 4.13 |
| 22:5n-6 | ***117 ± 1.93*** | | ***142 ± 3.27***** | 92.2 ± 3.56 | | 103 ± 5.29 | ***86.0 ± 2.63*** | | ***98.9 ± 3.98**** |
| N-6 PUFA | 3780 ± 54.3 | | 3931 ± 52.9 | 3405 ± 62.7 | | 3610 ± 130.4 | 3621 ± 55.4 | | 3534 ± 67.8 |
| 18:3n-3, ALA | 7.79 ± 0.72 | | 8.59 ± 1.00 | 2.43 ± 0.22 | | 2.06 ± 0.14 | 2.03 ± 0.13 | | 1.79 ± 0.13 |
| 18:4n-3 | ***2.18 ± 0.16*** | | ***4.31 ± 0.24***** | 2.26 ± 0.18 | | 2.85 ± 0.23 | ***1.63 ± 0.11*** | | ***2.94 ± 0.16***** |
| 20:3n-3 | ***0.82 ± 0.04*** | | ***1.08 ± 0.07**** | 0.78 ± 0.14 | | 0.80 ± 0.14 | 0.69 ± 0.09 | | 0.75 ± 0.13 |
| 20:4n-3 | ***2.07 ± 0.18*** | | ***3.37 ± 0.16***** | 1.04 ± 0.16 | | 0.90 ± 0.11 | 0.89 ± 0.13 | | 1.08 ± 0.28 |
| 20:5n-3, EPA | ***18.3 ± 0.57*** | | ***23.5 ± 0.96***** | ***102.3 ± 3.61*** | | ***124.4 ± 6.90**** | 7.71 ± 0.34 | | 9.03 ± 2.32 |
| 22:5n-3, DPAn-3 | | ***45.9 ± 0.97*** | ***56.6 ± 1.61***** | ***82.2 ± 2.13*** | | ***99.8 ± 5.15**** | 21.1 ± 1.26 | | 19.6 ± 1.13 |
| 22:6n-3, DHA | ***538 ± 8.22*** | | ***498 ± 9.26**** | 586 ± 12.90 | | 581 ± 24.69 | 765 ± 11.16 | | 764 ± 17.22 |
| N-3 PUFA | 615 ± 8.34 | | 595 ± 9.51 | 778 ± 13.57 | | 812 ± 26.15 | 799 ± 11.24 | | 799 ± 17.41 |
| Total PUFA | 4395 ± 54.9 | | 4527 ± 53.7 | 4182 ± 64.1 | | 4421 ± 133.0 | 4420 ± 56.5 | | 4333 ± 70.0 |
| Total FA | ***10027 ± 106.5*** | | ***10471 ± 111.9**** | ***9565 ± 135.2*** | | ***10260 ± 243.6**** | 10002 ± 120.8 | | 9910 ± 146.9 |

All values are expressed as means (nmol/g) ± SEM (pooled data, n = 28). Statistically significant differences relative to females within each diet group were determined by independent t-test, with ******* denoting a p-value < 0.05, and ******** a p-value < 0.001. ALA – α-linolenic acid; DHA – docosahexaenoic acid; DPAn-3 – docosapentaenoic acid; EPA – eicosapentaenoic acid; FA – fatty acid; MUFA – monounsaturated fatty acid; PUFA – polyunsaturated fatty acid; SFA – saturated fatty acid.

**Supplemental Table 8** – Red blood cell (RBC) percent weight of fatty acids in total fatty acids of ALA-, EPA- and DHA-fed female and male mice.

|  | RBC Relative Percent (Fatty Acid in % Total Fatty Acids) | | | | | | | | |
| --- | --- | --- | --- | --- | --- | --- | --- | --- | --- |
|  | **ALA-fed** | | | | **EPA-fed** | | | **DHA-fed** | |
| Fatty Acid | Females | | Males | Females | | Males | Females | | Males |
| 12:0 | 0.080 ± 0.011 | | 0.086 ± 0.015 | 0.048 ± 0.005 | | 0.039 ± 0.004 | ***0.047 ± 0.004*** | | ***0.036 ± 0.003**** |
| 14:0 | 0.482 ± 0.021 | | 0.488 ± 0.024 | 0.437 ± 0.025 | | 0.424 ± 0.021 | 0.427 ± 0.031 | | 0.372 ± 0.031 |
| 15:0 | ***0.053 ± 0.002*** | | ***0.061 ± 0.003**** | 0.053 ± 0.002 | | 0.058 ± 0.002 | 0.056 ± 0.002 | | 0.076 ± 0.021 |
| 16:0 | ***22.122 ± 0.277*** | | ***22.945 ± 0.281**** | 21.756 ± 0.356 | | 22.535 ± 0.264 | 21.979 ± 0.318 | | 22.458 ± 0.370 |
| 17:0 | ***0.165 ± 0.002*** | | ***0.183 ± 0.003***** | 0.171 ± 0.003 | | 0.175 ± 0.005 | 0.176 ± 0.003 | | 0.176 ± 0.004 |
| 18:0 | ***15.033 ± 0.122*** | | ***13.484 ± 0.138***** | ***14.452 ± 0.186*** | | ***13.506 ± 0.142***** | ***14.727 ± 0.123*** | | ***13.214 ± 0.127***** |
| 20:0 | ***0.199 ± 0.005*** | | ***0.265 ± 0.005***** | ***0.208 ± 0.006*** | | ***0.247 ± 0.008***** | ***0.197 ± 0.005*** | | ***0.260 ± 0.007***** |
| 22:0 | ***0.656 ± 0.024*** | | ***0.599 ± 0.013**** | 0.717 ± 0.022 | | 0.685 ± 0.024 | 0.705 ± 0.025 | | 0.721 ± 0.032 |
| 23:0 | 0.082 ± 0.004 | | 0.088 ± 0.003 | 0.099 ± 0.005 | | 0.098 ± 0.004 | ***0.088 ± 0.004*** | | ***0.110 ± 0.004***** |
| 24:0 | 1.016 ± 0.055 | | 0.992 ± 0.035 | 1.191 ± 0.065 | | 1.129 ± 0.065 | 1.149 ± 0.054 | | 1.183 ± 0.054 |
| SFAs | 39.888 ± 0.281 | | 39.191 ± 0.294 | 39.131 ± 0.310 | | 38.896 ± 0.304 | ***39.550 ± 0.258*** | | ***38.607 ± 0.297**** |
| 14:1 | 0.009 ± 0.001 | | 0.006 ± 0.001 | 0.005 ± 0.001 | | 0.008 ± 0.002 | 0.004 ± 0.001 | | 0.005 ± 0.001 |
| 16:1n-7 | ***0.567 ± 0.019*** | | ***0.774 ± 0.036***** | ***0.668 ± 0.030*** | | ***0.793 ± 0.028**** | ***0.503 ± 0.025*** | | ***0.716 ± 0.026***** |
| 16:1n-9 | ***0.080 ± 0.003*** | | ***0.094 ± 0.005**** | 0.099 ± 0.009 | | 0.109 ± 0.005 | ***0.072 ± 0.003*** | | ***0.097 ± 0.003***** |
| 18:1n-7 | ***1.560 ± 0.024*** | | ***1.886 ± 0.035***** | ***1.721 ± 0.030*** | | ***1.877 ± 0.037**** | ***1.449 ± 0.032*** | | ***1.757 ± 0.036***** |
| 18:1n-9 | ***9.140 ± 0.078*** | | ***9.802 ± 0.091***** | ***9.465 ± 0.098*** | | ***9.846 ± 0.088**** | ***8.746 ± 0.097*** | | ***9.404 ± 0.087***** |
| 20:1n-9 | ***0.260 ± 0.005*** | | ***0.349 ± 0.004***** | ***0.251 ± 0.011*** | | ***0.308 ± 0.008***** | ***0.243 ± 0.006*** | | ***0.314 ± 0.007***** |
| 22:1n-9 | 0.146 ± 0.016 | | 0.169 ± 0.019 | 0.136 ± 0.007 | | 0.168 ± 0.020 | 0.119 ± 0.007 | | 0.149 ± 0.016 |
| 24:1n-9 | 0.718 ± 0.034 | | 0.664 ± 0.025 | 0.832 ± 0.031 | | 0.797 ± 0.038 | 0.779 ± 0.030 | | 0.864 ± 0.032 |
| MUFAs | ***12.479 ± 0.105*** | | ***13.744 ± 0.131***** | ***13.178 ± 0.149*** | | ***13.906 ± 0.149**** | ***11.916 ± 0.129*** | | ***13.307 ± 0.131***** |
| 18:2n-6 | ***10.701 ± 0.175*** | | ***11.456 ± 0.138**** | 10.110 ± 0.207 | | 10.548 ± 0.191 | 10.614 ± 0.160 | | 10.692 ± 0.176 |
| 18:3n-6 | ***0.071 ± 0.002*** | | ***0.083 ± 0.003**** | ***0.063 ± 0.003*** | | ***0.073 ± 0.003**** | ***0.059 ± 0.002*** | | ***0.076 ± 0.003***** |
| 20:2n-6 | 0.234 ± 0.013 | | 0.260 ± 0.010 | 0.243 ± 0.016 | | 0.237 ± 0.010 | 0.238 ± 0.011 | | 0.240 ± 0.011 |
| 20:3n-6 | ***1.504 ± 0.024*** | | ***1.984 ± 0.023***** | ***1.474 ± 0.029*** | | ***1.807 ± 0.038***** | ***1.398 ± 0.028*** | | ***1.897 ± 0.039***** |
| 20:4n-6 | ***22.394 ± 0.153*** | | ***20.871 ± 0.170***** | ***21.271 ± 0.271*** | | ***20.064 ± 0.202***** | ***21.391 ± 0.186*** | | ***20.225 ± 0.195***** |
| 22:4n-6 | 2.505 ± 0.049 | | 2.441 ± 0.045 | 2.246 ± 0.045 | | 2.178 ± 0.048 | 2.039 ± 0.039 | | 2.028 ± 0.048 |
| 22:5n-6 | ***1.340 ± 0.035*** | | ***1.549 ± 0.043***** | 1.092 ± 0.029 | | 1.148 ± 0.031 | ***0.967 ± 0.027*** | | ***1.128 ± 0.037**** |
| N-6 PUFA | 38.749 ± 0.171 | | 38.643 ± 0.204 | 36.498 ± 0.224 | | 36.056 ± 0.184 | 36.705 ± 0.185 | | 36.286 ± 0.236 |
| 18:3n-3, ALA | 0.075 ± 0.007 | | 0.078 ± 0.009 | 0.024 ± 0.002 | | 0.019 ± 0.001 | 0.019 ± 0.001 | | 0.017 ± 0.001 |
| 18:4n-3 | ***0.021 ± 0.002*** | | ***0.039 ± 0.002***** | 0.022 ± 0.002 | | 0.026 ± 0.001 | ***0.015 ± 0.001*** | | ***0.028 ± 0.001***** |
| 20:3n-3 | ***0.009 ± 0.000*** | | ***0.011 ± 0.001**** | 0.009 ± 0.002 | | 0.009 ± 0.002 | 0.007 ± 0.001 | | 0.008 ± 0.002 |
| 20:4n-3 | ***0.022 ± 0.002*** | | ***0.034 ± 0.001***** | 0.011 ± 0.002 | | 0.010 ± 0.001 | 0.009 ± 0.001 | | 0.011 ± 0.003 |
| 20:5n-3, EPA | ***0.189 ± 0.004*** | | ***0.232 ± 0.007***** | ***1.105 ± 0.022*** | | ***1.260 ± 0.031***** | 0.079 ± 0.003 | | 0.091 ± 0.021 |
| 22:5n-3, DPAn-3 | | ***0.524 ± 0.017*** | ***0.616 ± 0.016***** | ***0.977 ± 0.020*** | | ***1.110 ± 0.025***** | 0.238 ± 0.015 | | 0.225 ± 0.013 |
| 22:6n-3, DHA | ***6.106 ± 0.163*** | | ***5.411 ± 0.141**** | ***6.941 ± 0.136*** | | ***6.474 ± 0.145**** | 8.566 ± 0.146 | | 8.689 ± 0.168 |
| N-3 PUFA | ***6.946 ± 0.181*** | | ***6.421 ± 0.155**** | 9.090 ± 0.160 | | 8.907 ± 0.170 | 8.933 ± 0.148 | | 9.069 ± 0.173 |
| Total PUFA | 45.694 ± 0.324 | | 45.063 ± 0.337 | 45.589 ± 0.360 | | 44.962 ± 0.326 | 45.638 ± 0.309 | | 45.355 ± 0.371 |
| Total FA | 34.570 ± 0.353 | | 33.114 ± 0.351 | 35.115 ± 0.393 | | 34.048 ± 0.330 | 34.685 ± 0.332 | | 34.292 ± 0.401 |

All values are expressed as % fatty acid in total RBC fatty acids ± SEM (pooled data, n = 28). Statistically significant differences relative to females within each diet group were determined by independent t-test, with ******* denoting a p-value < 0.05, and ******** a p-value < 0.001. ALA – α-linolenic acid; DHA – docosahexaenoic acid; DPAn-3 – docosapentaenoic acid; EPA – eicosapentaenoic acid; FA – fatty acid; MUFA – monounsaturated fatty acid; PUFA – polyunsaturated fatty acid; SFA – saturated fatty acid.

**Supplemental Table 9** – Brain DHA kinetics in ALA-, EPA- and DHA-fed male and female mice.

| Diet Group | Measured n-3 PUFA | Rate constant (*k*) (days^-1^ × 10^-3^) | | Half-life (*t_1/2_*) (days) | | Turnover rate (*J_out_*) (µmol/g/d) | |
| --- | --- | --- | --- | --- | --- | --- | --- |
|  |  | Female | Male | Female | Male | Female | Male |
| EPA-fed | DHA | n.d. ^#^ | 0.021 ± 0.004 | n.d. ^#^ | 33.0 ± 11.4 | n.d. ^#^ | 0.404 ± 0.08 |
| DHA-fed |  | 0.067 ± 0.011 | 0.058 ± 0.011 | 10.3 ± 1.9 | 12.0 ± 2.6 | 1.33 ± 0.22 | 1.13 ± 0.21 |

* represents statistically significant differences relative to females within each n-3 PUFA kinetic parameter, as determined by independent t-test, p-value < 0.05. ^#^ denotes kinetic values that could not be properly modeled by one-phase exponential decay resulting in a 95% interval and standard error that could not be determined. All values are expressed as means ± SEM (n = 4 mice per diet, per time point, per sex). ALA, α-linolenic acid; DHA, docosahexaenoic acid; DPAn-3, docosapentaenoic acid; EPA, eicosapentaenoic acid.


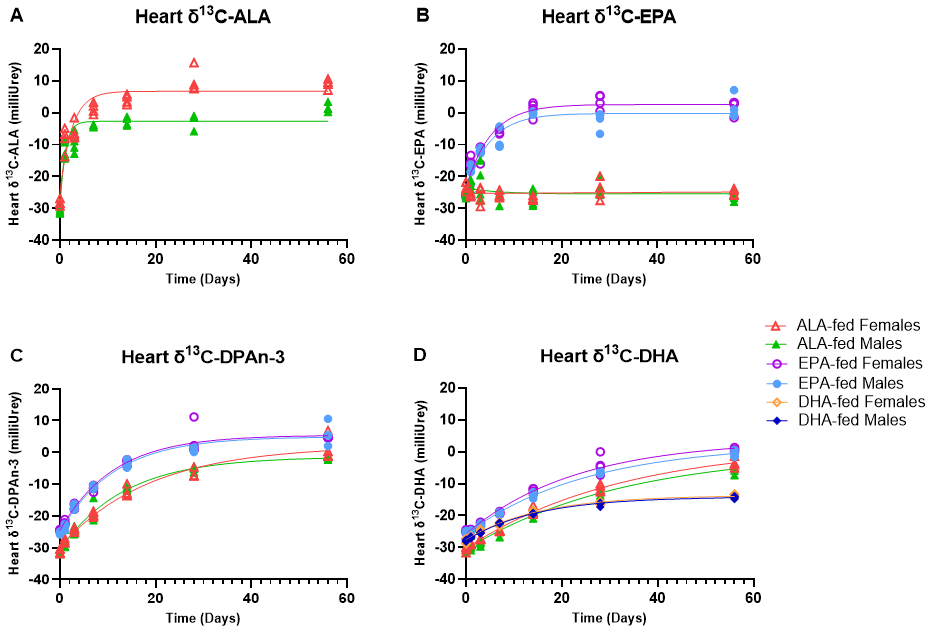


**Supplemental Figure 2** – Heart one-phase exponential decay dissociation curves for **(A)** δ^13^C-α-linolenic acid (ALA), **(B)** δ^13^C- eicosapentaenoic acid (EPA) and **(C)** δ^13^C- docosapentaenoic acid (DPAn-3) and **(D)** δ^13^C- docosahexaenoic acid (DHA). δ^13^C, carbon-13 content (milliUrey [mUr]).

**
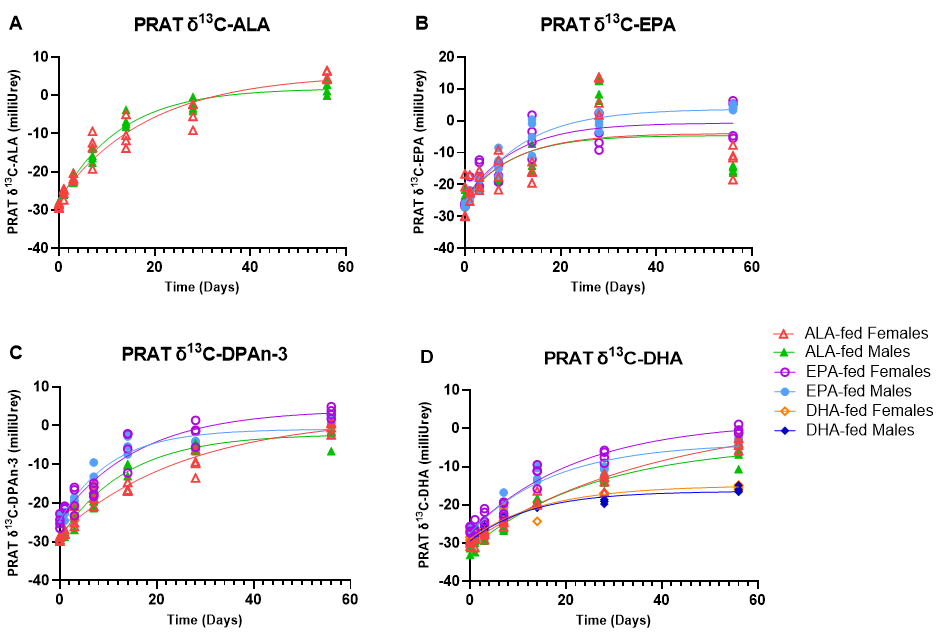
**

**Supplemental Figure 3** – Perirenal adipose tissue (PRAT) one-phase exponential decay dissociation curves for **(A)** δ^13^C-α-linolenic acid (ALA), **(B)** δ^13^C- eicosapentaenoic acid (EPA) and **(C)** δ^13^C- docosapentaenoic acid (DPAn-3) and **(D)** δ^13^C- docosahexaenoic acid (DHA). δ^13^C, carbon-13 content (milliUrey [mUr]).


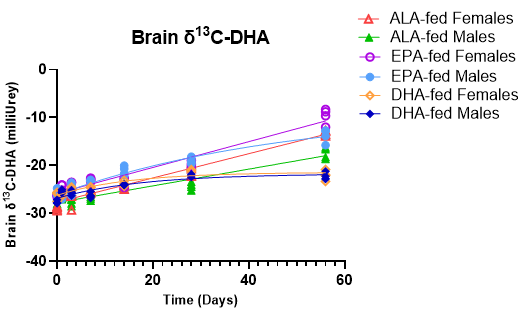


**Supplemental Figure 4** – Brain one-phase exponential decay dissociation curves δ^13^C- docosahexaenoic acid (DHA). δ^13^C, carbon-13 content (milliUrey [mUr]).


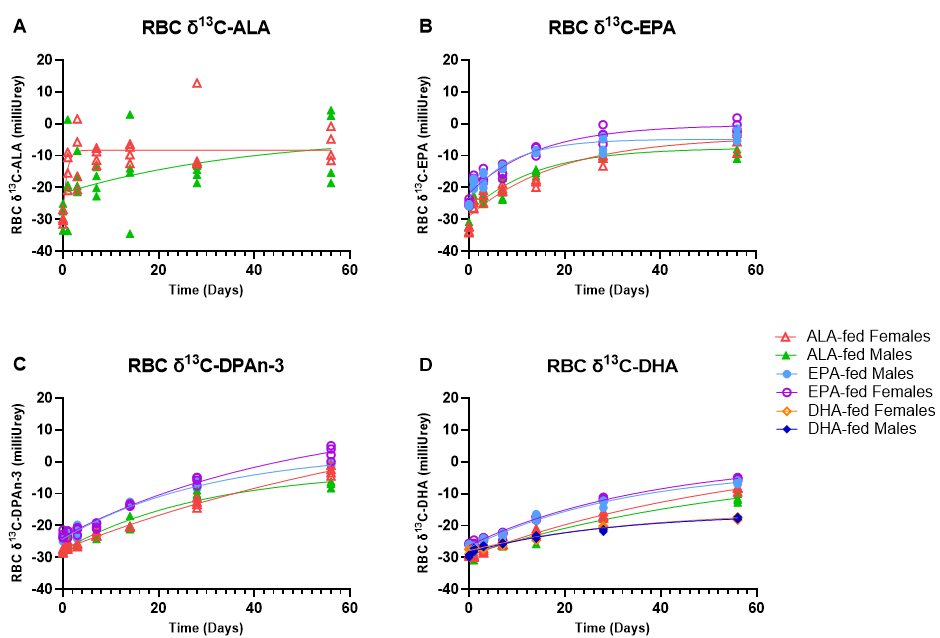


**Supplemental Figure 5** – Red blood cells (RBC) one-phase exponential decay dissociation curves for **(A)** δ^13^C-α-linolenic acid (ALA), **(B)** δ^13^C- eicosapentaenoic acid (EPA) and **(C)** δ^13^C- docosapentaenoic acid (DPAn-3) and **(D)** δ^13^C- docosahexaenoic acid (DHA). δ^13^C, carbon-13 content (milliUrey [mUr]).
